# A structural magnetic resonance imaging review of clinical motor outcomes from deep brain stimulation in movement disorders

**DOI:** 10.1093/braincomms/fcad171

**Published:** 2023-05-31

**Authors:** Luke Andrews, Simon S Keller, Jibril Osman-Farah, Antonella Macerollo

**Affiliations:** The Department of Pharmacology and Therapeutics, Institute of Systems, Molecular and Integrative Biology, University of Liverpool, Liverpool L3 9TA, UK; Department of Neurology and Neurosurgery, The Walton Centre NHS Foundation Trust, Liverpool L97LJ, UK; The Department of Pharmacology and Therapeutics, Institute of Systems, Molecular and Integrative Biology, University of Liverpool, Liverpool L3 9TA, UK; Department of Neurology and Neurosurgery, The Walton Centre NHS Foundation Trust, Liverpool L97LJ, UK; The Department of Pharmacology and Therapeutics, Institute of Systems, Molecular and Integrative Biology, University of Liverpool, Liverpool L3 9TA, UK; Department of Neurology and Neurosurgery, The Walton Centre NHS Foundation Trust, Liverpool L97LJ, UK

**Keywords:** MRI, DBS, Parkinson’s disease, dystonia, essential tremor

## Abstract

Patients with movement disorders treated by deep brain stimulation do not always achieve successful therapeutic alleviation of motor symptoms, even in cases where surgery is without complications. Magnetic resonance imaging (MRI) offers methods to investigate structural brain-related factors that may be predictive of clinical motor outcomes. This review aimed to identify features which have been associated with variability in clinical post-operative motor outcomes in patients with Parkinson’s disease, dystonia, and essential tremor from structural MRI modalities. We performed a literature search for articles published between 1 January 2000 and 1 April 2022 and identified 5197 articles. Following screening through our inclusion criteria, we identified 60 total studies (39 = Parkinson’s disease, 11 = dystonia syndromes and 10 = essential tremor). The review captured a range of structural MRI methods and analysis techniques used to identify factors related to clinical post-operative motor outcomes from deep brain stimulation. Morphometric markers, including volume and cortical thickness were commonly identified in studies focused on patients with Parkinson’s disease and dystonia syndromes. Reduced metrics in basal ganglia, sensorimotor and frontal regions showed frequent associations with reduced motor outcomes. Increased structural connectivity to subcortical nuclei, sensorimotor and frontal regions was also associated with greater motor outcomes. In patients with tremor, increased structural connectivity to the cerebellum and cortical motor regions showed high prevalence across studies for greater clinical motor outcomes. In addition, we highlight conceptual issues for studies assessing clinical response with structural MRI and discuss future approaches towards optimizing individualized therapeutic benefits. Although quantitative MRI markers are in their infancy for clinical purposes in movement disorder treatments, structural features obtained from MRI offer the powerful potential to identify candidates who are more likely to benefit from deep brain stimulation and provide insight into the complexity of disorder pathophysiology.

## Teaser

Andrews *et al*. conducted a systematic search into the studies utilizing structural MRI in association with or prediction of motor outcomes from deep brain stimulation in Parkinson’s disease, dystonia and tremor. Morphometric and diffusion-based approaches were identified as the most popular techniques, providing insight into localized and network-based changes across disorders.

## Graphical Abstract

**Graphical abstract:**
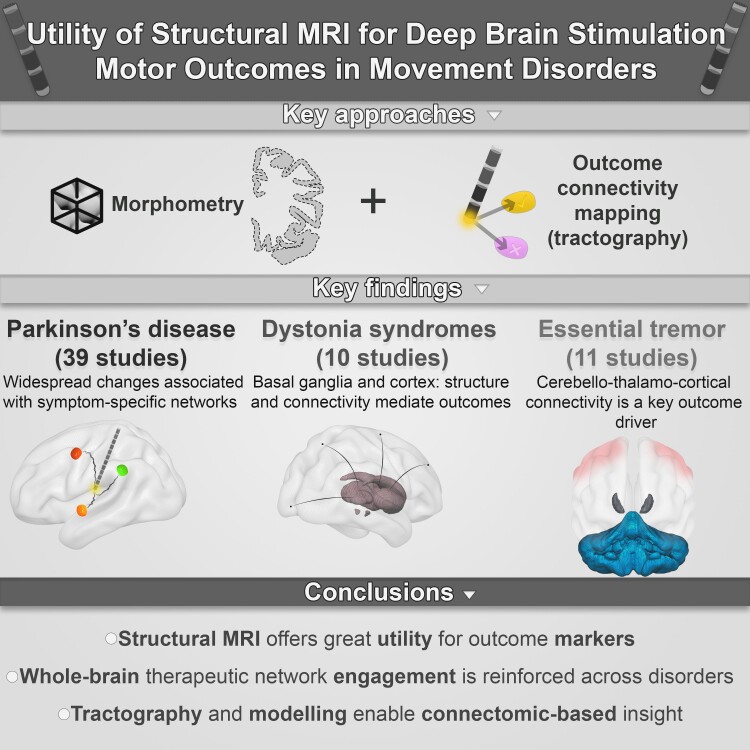

## Introduction

Deep brain stimulation (DBS) is a neurosurgical procedure used to treat a range of neurological and neuropsychiatric disorders. DBS has become the therapeutic option of choice for Parkinson’s disease, dystonia syndromes and essential tremor if pharmacological treatments fail to provide satisfactory therapeutic relief or induce significant unwanted side effects.^1,2^ Although DBS is a standard therapeutic choice in the context of clinical care pathways, satisfactory motor symptom improvement is not achieved for every patient and some patients can develop stimulation-related side effects.^3,4^ The accuracy of electrode positioning and variability in individualized DBS programming have been identified as key contributory factors driving outcome variability.^2^ Additionally, complications arising from the surgical procedure can lead to cases of sub-optimal motor outcomes.^5,6^ Importantly, even in cases with accurate electrode placement and no post-operative surgical contraindications, post-operative relief of motor symptoms can still be insufficient.^3,7^ This problem justifies a need to identify disorder-related, brain-based biomarkers that can be used to account for and predict improvements in clinical motor outcomes.

Mediating factors of motor outcomes that are unrelated to the neurosurgical procedure have been explored. Information derivable from routine imaging for DBS screening, pre-operative stereotactic planning, and follow-up assessment present avenues to explore factors associated with motor outcome variability. Pre-operative T1- and T2-weighted structural magnetic resonance imaging (MRI) and post-operative MRI-computed tomography (CT) fusion are acquired for each patient as standard. Additional sequences that have been predominantly constrained to research applications are being increasingly incorporated into centres’ imaging routines. One modality is diffusion-weighted MRI (dMRI) which can provide clinical utility by aiding electrode implantation accuracy in relation to white matter tract bundles.^8,9^ Together, these sequences can characterize pre-and post-operative structural brain-related information which can be related to changes in clinical motor symptom scores.

Identifying brain-related features or biomarkers for DBS motor outcomes is crucial for several reasons. Most importantly, determining patient-specific outcome-related factors will inform the optimization of therapeutic benefits and reduce potential surgery-related side effects.^10^ Identifying individualized prognostic markers may provide Supplementary Information for optimal candidate selection, develop individual programming processes, and manage patient expectations. Additionally, such research offers the potential to address questions that are poorly understood. Addressing outcome variability from DBS may help to identify mechanisms pertaining to disorder-related pathology and shed light on the processes underpinning DBS’ therapeutic modulatory effects.

This study aimed to review the literature surrounding the use of structural MRI to identify features that have been used to account for variability in motor outcomes following DBS. Importantly, other imaging domains such as functional MRI, used to measure regional haemodynamic brain changes, have also shown efficacy to provide markers for motor outcomes.^11‐14^ Crucially, however, functional MRI is not globally acquired as part of routine imaging protocols for DBS. Therefore, this review sought to exclude these studies and focus on techniques that are currently embedded into the clinical evaluation pathway for patients undergoing DBS surgery. Studies focusing on efficacy comparisons of DBS targets were excluded given the lack of focus on structural MRI application and existing comprehensive literature.^15‐17^ Additionally, quantification of stimulation overlap or measurement of contacts relative to the defined neurosurgical target were excluded, given that accuracy is indicative of successful treatment and is reviewed elsewhere.^18‐20^

## Methods

### Search protocol

This review was conducted in accordance with the preferred reporting items for systematic reviews and meta-analyses (PRISMA) statement.^21,22^ PubMed, Scopus, and Web of Science electronic databases were used to identify relevant literature. Article searches were restricted to full texts, written in English with a date of publication between 1 January 2000 and 1 April 2022. Database search terms are included as Supplementary Information.

### Literature eligibility

To identify candidate articles and discount topics of non-interest, titles were scanned by the primary author. Following initial selection, the resultant articles were subject to abstract and full-text review, based on inclusion criteria (Table 1). All studies were required to satisfy all points of the inclusion criteria to be included in the current review. In line with PRISMA guidelines, included articles reference lists were searched to identify additional literature. The final selection of article relevancy was confirmed by A.M.

**Table 1:** Inclusion criteria for the systematic review

Inclusion criteria questions
(1) The article must clearly relate DBS with motor outcomes (excluding speech symptoms)
(2) Structural MRI must be related to DBS motor outcomes
(3) Structural MRI must be used to account for an empirical neurological feature, unrelated to procedure accuracy, neurological target comparison, procedural side effects (i.e. micro-lesion effects) or DBS programming parameters
(4) The clinical population must be adult patients with either Parkinson’s disease, dystonia syndromes or tremor
(5) The article must not present a case study or literature review

To summarize the studies included in the final selection, information was recorded by L.A. Author(s), publication date, patient sample size included in the imaging analyses, structural MRI data and analysis method, motor outcome scale, motor follow-up period and main findings were recorded for each study.

## Results

### Search strategy

**Figure 1:**
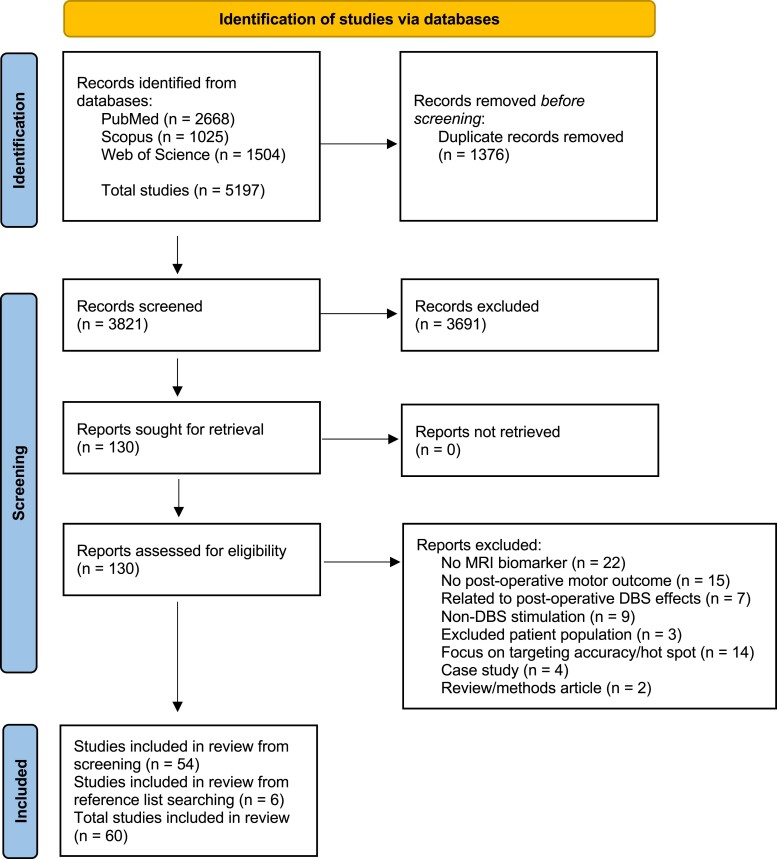
**The strategy used in the current review.** PRISMA flow diagram outlining study identification, screening and inclusion steps are presented. Abbreviations: *n*, number of studies

The search yielded a total of 5197 and 3821 articles pre-and-post de-duplication, respectively. Following title screening, 130 articles underwent full-text review using the inclusion criteria. A total of 54 articles were extracted from the search to be included in the systematic review. Six additional articles were extracted from additional sources. Herein, 60 studies were included in the final review (the article selection process is presented in Fig. 1).

### Data extraction

Of the 60 identified studies, 39 studies focused on patients with Parkinson’s disease,^23‐61^ 10 studies focused on patients with dystonia syndromes,^62‐71^ and 11 studies focused on patients with essential tremor (and additional tremor subtypes).^72‐82^ Information regarding authors, study sample sizes (n), movement disorder diagnosis, targeted DBS region, main structural MRI technique utilized (MRI), clinical scale, post-operative follow-up period(s) and summarized main findings are presented as supplementary material (see Supplementary Table 1). Note, the main findings summary is in relation to overall motor improvement via the reported clinical motor scale, unless otherwise stated.

### Study characteristics

Across the 60 included studies, a total of 2202 patients who had undergone DBS were included in this review. A total of 1693 patients were diagnosed with Parkinson’s disease (108 of these patients were reported as an explicitly defined clinical subtype: 24 akinetic-rigid subtype, eight postural instability gait disorder subtype, 72 Parkinson’s disease with camptocormia and six with tremor-dominant Parkinson’s disease subtype); 279 patients were diagnosed with dystonia syndromes (16 with X-linked dystonia-parkinsonism, 20 with dystonic tremor and 2 with myoclonic dystonic tremor); 230 patients were diagnosed with essential tremor.

A total of nine scales used for measuring motor outcome improvement following DBS were identified in this study. Clinical scales used to measure cardinal symptoms of Parkinson’s disease including the unified Parkinson’s disease rating scale (UPDRS III)^83^ observed in 30 studies.^23‐26,28,30,32‐39,42,44‐48,50‐59^ The Movement Disorder Society-Sponsored Revision of the UPDRS (MDS-UPDRS III)^84^ was used in eight studies.^27,29,31,40,41,43,60,61^ The gait and falls questionnaire (GFQ), freezing of gait questionnaire (FoGQ) and a stepping-in-place task (SIP), were used to assess axial symptoms in patients with Parkinson’s disease.^49,57^ For generalized, focal, segmental dystonia and X-linked dystonia-Parkinsonism, the Burke–Fahn–Marsden dystonia rating scale (BFMDRS)^85^ was administered in eight studies.^62‐65,68‐71^ In addition, the unified dystonia rating scale (UDRS)^86^ was administered for generalized dystonia in one study.^66^ For cervical dystonia, the Toronto Western spasmodic torticollis rating scale (TWSTRS)^87^ was administered in three studies.^63,65,67^ For tremor, the Fahn–Tolosa–Marin (FTM) clinical rating scale for tremor^88^ was used in nine studies.^72,74‐80,82^ In two studies, clinical testing for motor symptoms was not assessed using standardized clinical scales.^73,81^

## Reporting clinical outcomes from deep brain stimulation

The process to determine group-based clinical post-operative motor improvements were not standardized across studies. The most common approach was to dichotomize patient groups using cut-off thresholds of clinical improvements pre-and-post DBS surgery. These thresholds are summarized in Table 2. From here on, patients reported as having non-satisfactory clinical motor outcomes are referred to as sub-optimal responders, and patients classified as having satisfactory clinical motor outcomes are referred to as optimal responders. Other grouping approaches included dichotomizing patients based on the presence of MRI signal abnormalities,^64^ DBS side effects^53^ and contacts connected to a defined number of regions of interest.^54^ Details can be found in Table 3.

**Table 2:** Post-operative DBS motor response grouping thresholds

Author(s)	Grouping threshold(s)—pre-and-post change^a^
Parkinson’s disease	
Price *et al.*, 2011^48^	2-standard error of mean change (21-point improvement) for patients who ‘improved’
Chen *et al.*, 2018^28^	>50%—‘Group I’, ≤ 50%—‘Group II’ (for symptom improvement)
Strotzer *et al.*, 2019^50^	≥50%—‘effective contact’, <50%—‘noneffective contact’
Younce *et al.*, 2019^59^	30% UPDRS III reduction—‘desirable response’
Erdogan *et al.*, 2020^30^	≥30%—‘good outcome’
Hamed *et al.*, 2020^34^	≥20%—‘good outcome subgroup’, <20%—‘poor outcome subgroup’
Yim *et al.*, 2020^58^	≥70%—‘higher motor improvement’, <70%—‘lower motor improvement’
Abdulbaki *et al.*, 2021^23^	31.88% ± 8.64%—‘tremor responders’, 28.73% ± 18.4%—‘tremor non-responders’
Liu *et al.*, 2021^43^	≥30% ‘optimal outcome’, <30%—‘sub-optimal outcome’
Li *et al.*, 2022^60^	>25%—‘responders’, <25%—‘non-responders’
Dystonia syndromes	
Vasques *et al.*, 2009^71^	97 ± 4.6%—‘responded well’, 56.9 ± 6%—‘less improvement’
Fečíková *et al.*, 2018^63^	>50%—‘good responders’, 25–50%—‘partial responders’, <25%—‘non-responders’
Gonzalez-Escamilla *et al.*, 2019^65^	<70%—‘moderate outcome group’, >70%—‘superior outcome group’
Okromelidze *et al.*, 2020^66^	>25%—‘responders’, ≤ 25%—non-responders
Tremor	
Coenen *et al.*, 2014^74^	<60%—‘moderate tremor control’, >60% ‘excellent tremor control’

a Where reported, percent change scores are reported as mean ± standard deviation.

**Table 3:** Regions associated with outcomes using diffusion MRI in Parkinson’s disease

Study	Seed region	Areas associated with positive outcomes	Areas associated with negative outcomes	Symptoms
Vanegas-Arroyave *et al.*, 2016^54^	STN	SFG and thalamus^a^		UPDRS III^a^
Akram *et al.*, 2017^24^	STN	bilateral M1^d^, SMA^b,c^, PFC^b^		Rigidity^b^; bradykinesia^c^; tremor^d^
Horn *et al.*, 2017^35^	STN and cZi	SMA, SFG and cerebellum^a^		UPDRS III^a^
Krishna *et al.*, 2019^39^	STN	Left DLPFC (8BL, 9a, 9p, SFL), left PMC (6ma, 6mp)^b^; right DLPFC (8BL, 9 m, 9a, SFL), right M1 (4)^c^; bilateral DLPFC (8BL, 9p, SFL), bilateral PMC/SMA (6ma, 6mp), right M1 (4)^d^	right frontal polar cortex (a/p-47r), right DLPFC (a/p9–46v, 10d, 10pp)^e^	rigidity^b^; bradykinesia^c^, tremor^d^, side effects (paresthesia/motor contractions)^e^
Strotzer *et al.*, 2019^50^	STN	ML, BIC, TF and IML^b^; i-DN, i-SCP, FAL, m-ALIC and MFB^c^; Zi and PPN^c,d^; GPe^d^	GPi and IFG (BA45/46)^b^; central pons, c-MCP, SPL and iM1^c^; M1^d^	rigidity^b^; bradykinesia^c^; tremor^d^
Treu *et al.*, 2020^52^	STN	PMv and pre-SMA^a^	M1 and S1^a^	UPDRS III^a^
Vassal *et al.*, 2020^55^	STN	Brainstem tegmentum, SCP, contralateral cerebellum, SMA, LPMC and M1^a^		UPDRS III^a^
Wang *et al.*, 2021^56^	STN	Pre-SMA, ACC, dmPFC^a^	M1 and S1^a^	UPDRS III^a^
Lai *et al.*, 2021^40^	GPi	Right S1^f^		camptocormia^f^
Lai *et al.*, 2021^41^	STN	Right SMA, right PMd, bilateral PMv and right pre-SMA^f^		camptocormia^f^
Raghu *et al.*, 2021^49^	PPN	PCG^g,h^; S1 (BA1, BA2)^g^	SCP^g,h^; SMA^h^	GFQ^g^; FoGQ^h^
Tsuboi *et al.*, 2021^53^	Pallidal VTA	S1, cerebellum, STN, VOa and VOp^a^	SMA/PMC and associative cortex^a^	SID^I^
Chen *et al.*, 2022^29^	STN	SMA and precentral cortex^a^	right SFC, M-FC, IFC, I-OFC, M-OFC, S-OFC, ACC, MC and caudate^a^	UPDRS III^a^
Gonzalez-Escamilla *et al.*, 2022^32^	STN	M1 and SMA^a^		UPDRS III^a^

a Associated with global UPDRS III outcomes.

b Associated with rigidity outcomes.

c Associated with bradykinesia outcomes.

d Associated with tremor outcomes.

e Associated with paraesthesia and motor contraction side effects.

f Associated with camptocormia.

g Associated with gait and falls.

h Associated with freezing of gait.

i Associated with SID.

Abbreviations: ACC, anterior cingulate cortex; FAL, anterolateral fasciculus; BIC, brachium of the inferior colliculus; BA, Brodmann area; cZi, caudal zona incerta; c-MCP, contralateral middle cerebellar peduncle; DLPFC, dorsolateral prefrontal cortex; dmPFC, dorsomedial prefrontal cortex; FoGQ, freezing of gait questionnaire; GFQ, gait and falls questionnaire; GPe, globus pallidus externus; GPi, globus pallidus internus; IFC, inferior frontal cortex; IFG, inferior frontal gyrus; I-OFC, inferior orbitofrontal cortex; IML, internal medullary lamina of the thalamus; i-SCP, ipsilateral superior cerebellar peduncle; iM1, ipsilateral primary motor cortex; iPMC, ipsilateral premotor cortex; LPMC, lateral premotor cortex; MFB, medial forebrain bundle; ML, medial lemniscus; m-ALIC, medial part of the anterior limb of the internal capsule; MPFC, medial prefrontal cortex; M-FC, middle frontal cortex; MFG, middle frontal gyrus; M-OFC, middle orbitofrontal cortex; OFC, orbitofrontal cortex; PPN, pedunculopontine nucleus; PFC, prefrontal cortex; PMC, premotor cortex; PMd, premotor dorsal; PMv, premotor ventral; M1, primary motor cortex; S1, primary somatosensory cortex; STN, subthalamic nucleus; SCP, superior cerebellar peduncle; SFC, superior frontal cortex; SFG, superior frontal gyrus; SFL, superior frontal language area; S-OFC, superior orbitofrontal cortex; SPL, superior parietal lobe; SMA, supplementary motor area; TF, thalamic fasciculus; UPDRS, Unified Parkinson’s Disease Rating Scale; VOa, ventralis oralis anterior; VOp, ventralis oralis posterior; VTA, volume of tissue activated

### Identified MRI techniques

Several MRI methods and analysis techniques were identified from the 60 included studies and could be broadly characterized based on a focus on brain morphometry, iron-related features and dMRI approaches.

#### Manual image inspection

The most rudimentary approach identified in the review was manual image inspection for features associated with pathology. A total of three studies quantified image features in a manual fashion.^27,30,64^

#### Brain morphometry

**Figure 2:**
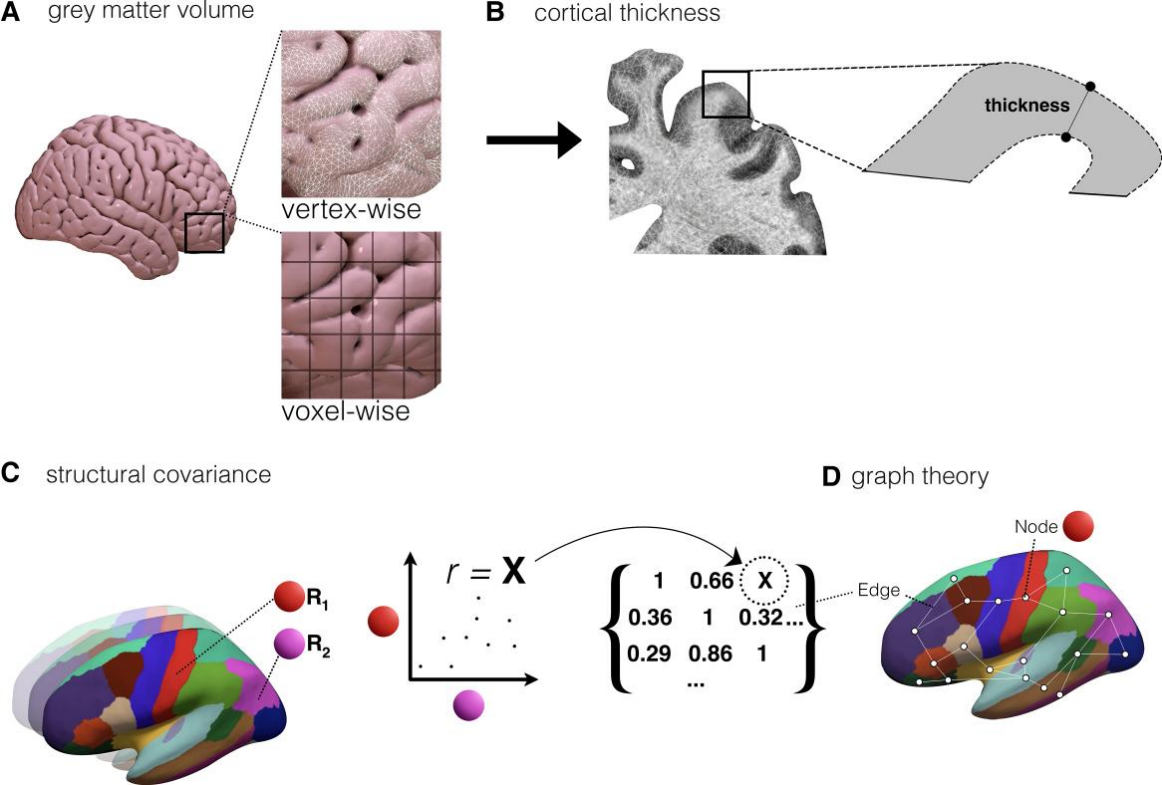
**Summary outline of morphometric approaches identified in the current review.** (**A**) GMv can be quantified either in a vertex (using FreeSurfer) or voxel-wise manner (using voxel-based morphometry). (**B**) Cortical thickness can be quantified in a vertex-wise manner (using FreeSurfer). (**C**) Structural covariance using two regions of interest. A correlation between two regions using a metric (such as cortical thickness) forms the entry of a covariance matrix which can be used to define the edge in a graph theory approach between given regions (**D**). Abbreviations: cortical thickness, CT; grey matter volume, GMv

Several computational methods were used to quantify brain morphometry (Fig. 2). Four studies utilized semi-automated approaches, requiring manual delineation of the brain region before an automatic reconstruction.^26,48,70,71^ A total of 17 studies utilized fully automated approaches to segment and process images for morphometric quantification. Seven studies adopted a voxel-based morphometry approach which is a fully automated technique used to identify voxel-wise changes of grey and white matter between groups (Fig. 2A).^28,31,32,53,54,56,57^

The most used software in this review was FreeSurfer (https://surfer.nmr.mgh.harvard.edu), an open-source, widely adopted automated pipeline, for automatic segmentation, reconstruction and parcellation of structural MRI, used in eight of the studies (Fig. 2A).^31,32,45,46,49,59,61,65^ FreeSurfer enables calculation of various structural parameters of cortical grey and white matter via whole-brain, vertex-wise analysis and also a volume of subcortical grey matter. Cortical thickness, computed as the distance between vertices on the pial surface to grey-and-white matter boundary surface vertices,^89^ was a feature used to associate brain regions with outcomes from DBS (Fig. 2B).

One study used cortical thickness obtained from FreeSurfer to create ‘structural covariance’ networks (Fig. 2C).^65^ Structural covariance networks have been utilized as a theoretical construction of the structural connectome, whereby regions that share similar structural properties, ascertain to some degree, shared connectivity.^90‐92^ The authors then analysed the networks using a graph theoretical app, a mathematical approach that is used to infer topological network metrics such as efficiency and organization.^93^

In-house medical imaging software was used in two studies. Namely, NeuroQuant (https://www.cortechs.ai/neuroquant/) 58 and Zero-Download Ambassador picture archiving and communication system software (DR Systems, Inc., San Diego, California, USA).^34^

#### Iron proxy assessment

MRI methods were used to assess proxies for iron levels in two studies focused on patients with Parkinson’s disease. One study used quantitative susceptibility mapping to identify radiomic features from patient MRI.^43^ Quantitative susceptibility mapping is a technique that is sensitive to the magnetic susceptibility of tissue and as such, can determine changes associated with the paramagnetic properties indicative of iron deposition.^94,95^ Another study measured T2 relaxation times from multi-contrast spin-echo sequences.^44^ T2 relaxation time has been observed to shorten in the presence of iron concentration and as such, can be used as a measurement of iron deposition.^96^

#### Diffusion imaging

**Figure 3:**
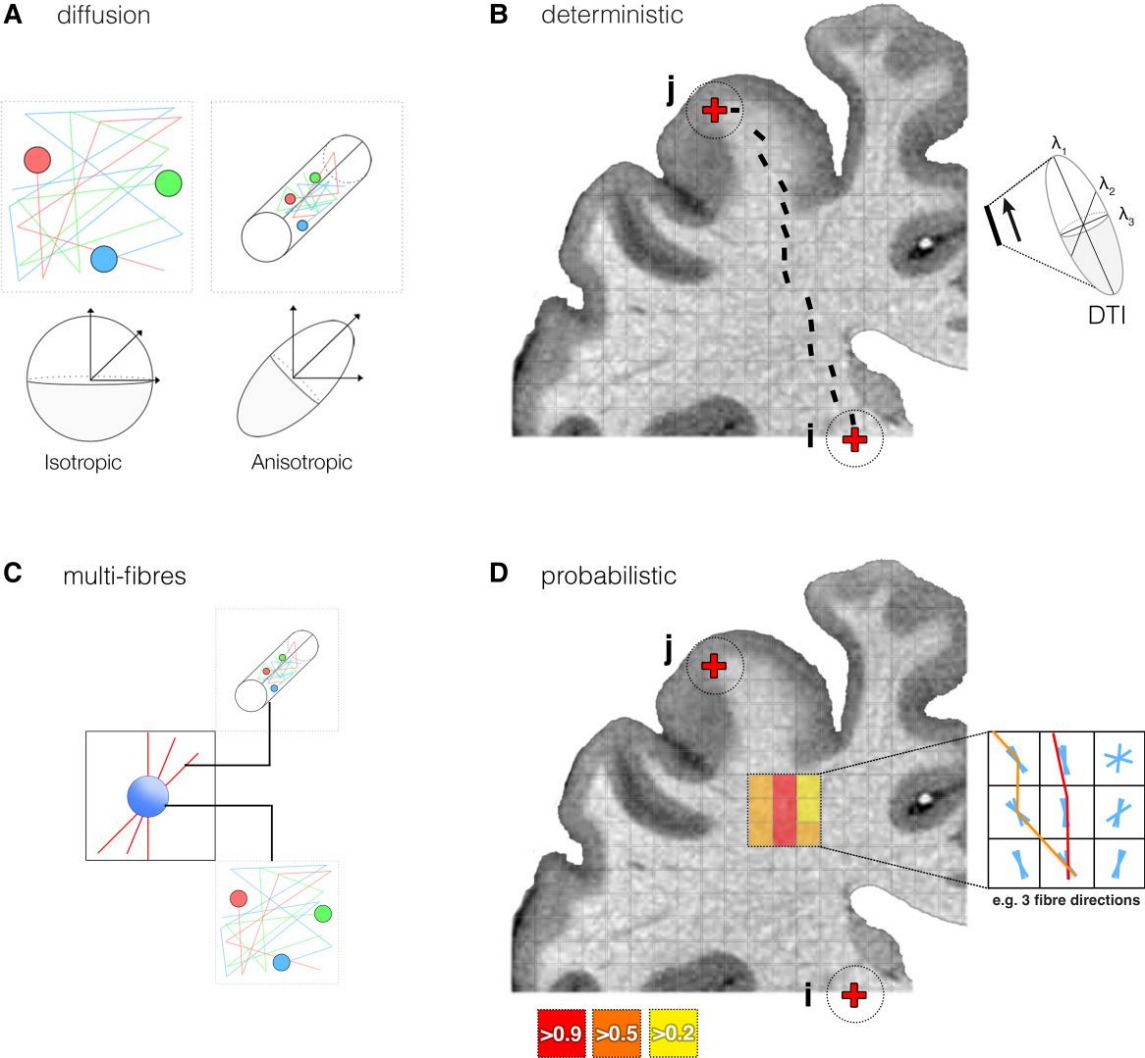
**Diffusion modelling and tractography.** (**A**) In unrestricted environments, diffusion is free in all directions, represented by the spheroid (isotropic); in restricted environments, such as along axons, diffusion has preferred orientation, represented by the prolate spheroid (anisotropic). (**B**) A schematic of deterministic streamline creation [between points (i) and (ii)], whereby each step follows the principal eigenvector (denoted with λ_1_) of the modelled diffusion tensor (right). (**C**) The ball-and-stick approach (identified as the most common approach to multi-fibre modelling) separates intra-axonal (anisotropic; stick) and outer-axonal (isotropic; ball) diffusion compartments to attribute voxel-wise signal to restricted and free water diffusion, respectively.^97,98^ (**D**) Probabilistic tractography repeats streamline propagation from point *i* to point *j*, following the orientation trajectories that best model the acquired diffusion signal. The probability of a possible connection between *i* and *j* is a ratio of streamlines intersecting *j* given the total intersecting *i* (total streamlines propagated)

dMRI was the most common approach, identified in 37 studies.^23‐25,28,29,32,35,38‐42,47,49‐56,60,66‐69,72‐82^ dMRI is sensitive to the motion of water diffusion in tissue and can capture diffusion restriction associated with the hydrophobic barriers of myelinated axons (Fig. 3A).^99,100^ Diffusion in such cases is said to be anisotropic, travelling preferably along white matter pathways, and can be used to infer the geometrical orientation of white matter at a voxel-wise level. A tractography is a post-processing approach that is applied to diffusion imaging and enables the reconstructive modelling of possible white matter bundles (or streamlines) in vivo.^101^

The simplest approach to tractography identified in this review was deterministic diffusion tensor imaging (DTI),^28,42,47,51,55,73,74,81^ which models a single orientation of voxel-wise diffusion using a tensor (Fig. 3B).^102,103^ The tensor reflects the maximum likelihood pathway through the DTI data, and thus, the algorithm steps in the direction of the modelled tensor’s principal eigenvector (or fastest orientation axis of diffusion) per voxel.^97^ The fundamental issue with tensor models is that given the minute scale of estimated fibres (∼0.16 to 9 μm) relative to typical voxel sizes (∼1 to 3 mm^3^), orientations are seldom coherent, and so single-direction tensors are an oversimplified model of the local microstructural geometrical organization.^104^ Furthermore, due to measurement noise and orientation complexity in dMRI, uncertainty is not accounted for in deterministic approaches.

Multi-fibre modelling is used to better estimate multiple diffusion directions, and thus, better represent the complex geometrical organization of fibres in each voxel. The most common multi-fibre approach here was using a fibre Orientation Distribution Function implemented as part of FSL (FMRIB Software Library; https://fsl.fmrib.ox.ac.uk/fsl; Fig. 3C).^97,98^ Multi-fibre modelling was combined with probabilistic tractography in 13 studies.^23,24,38,39,49,50,60,67,69,76,77,79,80^ Probabilistic tractography quantifies the probability of each voxel being intersected from seed over successive sampling runs.^97,105^ Areas of uncertainty are reflected by voxels with corresponding low probabilities and thus, reflect confidence bounds around the most probable connections. Probabilistic multi-fibre approaches offer a more conservative approach when modelling non-dominant connectivity pathways.^97^

Aside from patient-specific dMRI, this review highlighted the use of structural normative connectomes in 13 studies.^25,29,35,40,41,52,53,56,66,72,75,78,82^ Structural normative connectomes are the aggregation of multiple individual tractograms or whole-brain tractography images.^35,56,106,107^ Connectomes were constructed from both healthy samples and ‘disease-matched’ cohorts and were used to enable an analysis of 658 patients (averaging 50.6 patients per study), presented with relation to each study in Table 4. A total of 24 studies identified in this review acquired patient-specific dMRI, totalling 421 patients for analysis (averaging 17.5 patients per study) (Table 4).

**Table 4:** Normative connectomes used for tractography analyses

Authors	Connectome	*n* ^a^
Parkinson’s disease		
Horn *et al.*, 2017^35^	PPMI^b^	90
Avecillas-Chasin and Honey, 2020^25^	PPMI^b^	43
Treu *et al.*, 2020^52^	PPMI-85^c^	85
Lai *et al.*, 2021^40^	HCP-32^d^	32
Lai *et al.*, 2021^41^	PPMI-85^c^	85
Tsuboi *et al.*, 2021^53^	HCP^b^	30
Wang *et al.*, 2021^56^	− PPMI-85^c^	− 85
	− HCP-32^d^	− 32
Chen *et al.*, 2022^29^	PPMI-85^c^	85
Dystonia syndromes		
Okromelidze *et al.*, 2020^66^	HCP-32^d^	32
Tremor		
Al-Fatly *et al.*, 2019^72^	healthy subjects^b^	20
Dembek *et al.*, 2020^75^	HCP-32^d^	32
Middlebrooks *et al.*, 2021^78^	HCP^b^	124
Tsuboi *et al.*, 2021^82^	HCP^b^	125

a *n* represents the sample size used to construct the connectome, and not the number of patients included as part of each study.

b The normative connectome is uniquely used within the given study.

c The PPMI imaging normative connectome obtained from 85 patients with Parkinson’s disease.

d The MGH-USC HCP normative connectome obtained from 32 healthy adult subjects. Abbreviations: HCP, human connectome project; MGH-USC, Massachusetts General Hospital—University of Southern California; NC, normative connectome; PPMI, Parkinson’s progression markers initiative.

**Figure 4:**
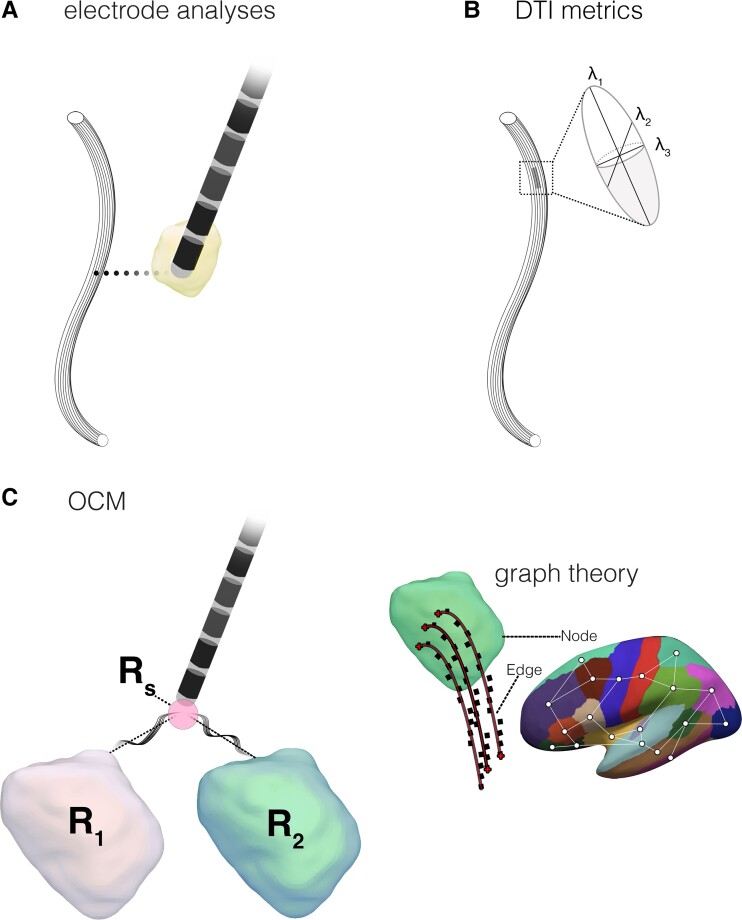
**Summary outline of diffusion techniques identified in the current review.** (**A**) Tracts reconstructed by tractography can be related to the VTA, or proximity to the electrode. (**B**) Quantitative metrics can be extrapolated from tracts by computations on tensor eigenvalues (λ) to provide insight into microstructure. (**C**) Streamline counts modelled from a seed region of interest or VTA activation (*R_s_*) to other regions (*R*_1_, *R*_2_) can be correlated with clinical outcome changes. The number of streamlines intersecting a region of interest can be used as a proxy of connectivity ‘strength’ and can be used to define the edge in graph theory. Abbreviations: outcome connectivity mapping, OCM

The analytical approaches applied to dMRI could be split into three notable categories in this review. Reconstructive modelling of the DBS contact’s electric field, termed the volume of tissue activation (VTA), using MRI-CT fusion overlaid onto, and related to tract reconstruction was a methodological approach (Fig. 4A).^108^ A total of three studies quantified the overlap of VTA with specific neural regions or tracts of interest.^23,28,47,51,60,69,73‐75,81^ A similar method used in 10 studies measured the distance (or proximity) of reconstructed contacts/VTA to specific tracts.^23,28,47,51,60,69,73‐75,81^ Another method involved assessing quantitative diffusion metrics from tensor-reconstructed tracts, identified in three studies (Fig. 4B).^32,42,49^ Diffusion metrics are commonly used as proxies for ‘microstructure’ given that they quantify variability in diffusion properties and can be used to infer pathological alterations.^109,110^ A final method involved correlating the number of intersecting streamlines between seeds at patient-specific VTA (or implanted brain region) to other regions in relation to clinical outcomes (Fig. 4C, left). Terminating regions could be pre-defined targets of interest, or as a *post hoc*, whole-brain assessment. Streamline-based intersection is a widespread approach exploreing ‘structural connectivity’ between brain regions.^24,29,32,35,39‐41,49,50,52‐56,66‐68,72,76‐78,80,82^ In this review we refer to this approach as ‘outcome connectivity mapping (OCM)’, in which 23 studies were included.^24,29,32,35,39‐41,49,50,52‐56,66‐68,72,76‐78,80,82^ Importantly, these studies did not measure overlap, proximity, or connectivity to the neurosurgical target (as outlined as part of the inclusion criteria). One study applied graph theory, using the number of streamlines intersecting each region of interest as the network connectivity measure (Fig. 4C, right).^38^

## Structural MRI applications

### Parkinson’s disease

#### Morphometric findings

**Figure 5:**
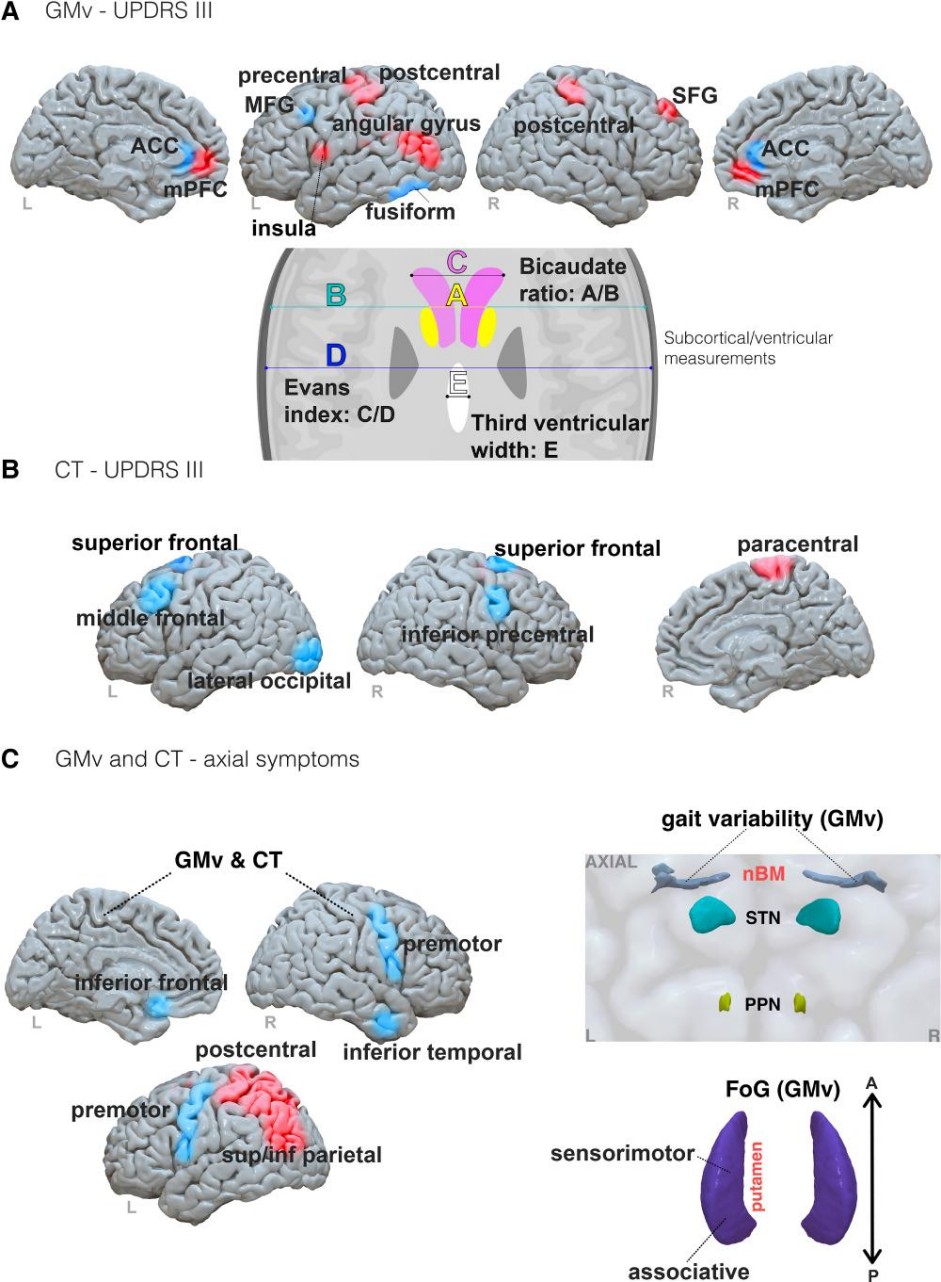
**Results summary of morphometric results from identified Parkinson’s disease studies.** (**A**) Top: brain regions identified in GMv associations with UPDRS III outcomes. Bottom: A schematic representation of ventricular measurements identified in the current review (**B**) A visual summary of regions identified in cortical thickness associations with UPDRS III outcomes. (**C**) Left: brain regions identified through GMv and cortical thickness associations with axial symptoms. Right: an axial representation of the PPN and nucleus basalis of Meynert in relation to the STN. Regions associated with symptom improvement and worsening are indicated in blue and red, respectively. Abbreviations: ACC, anterior cingulate cortex; CT, cortical thickness; FoG, freezing of gait; GMv, grey matter volume; MFG, middle frontal gyrus; mPFC, medial prefrontal cortex; nBM, nucleus basalis of Meynert; PPN, pedunculopontine nucleus; SFG, superior frontal gyrus; STN, subthalamic nucleus

A total of 18 studies used morphometric approaches (a visual summary of findings is presented in Fig. 5). Note, for all results figures, brain regions are reported in line with the nomenclature used in the study and are spatially approximated based on the respective study figures and/or atlases.

In patients with Parkinson’s disease, manual inspection approaches identified no significant association of lacunar vascular changes,^30^ but a significant effect of global white matter hyperintensity presence on poorer post-operative UPDRS III outcomes.^27^

With regards to voxel-based morphometry, a positive association of global white matter volume with UPDRS III improvements in a group of 21 patients with idiopathic Parkinson’s disease was reported.^58^ In 81 patients with Parkinson’s disease, larger grey matter volume (GMv) clusters in the left fusiform and middle frontal gyrus (MFG), bilateral anterior cingulate cortex and reduced volumes in the left insula, left pre-and-post central regions and right putamen volumes were reported in patients with satisfactory motor response to DBS.^58^ More recently, reduced GMv clusters in the bilateral medial prefrontal cortex (PFC), left angular gyrus, right superior frontal gyrus (SFG) and left MFG were associated with unsatisfactory UPDRS III outcomes.^36^ Two voxel-based morphometry studies focused on gait-related Parkinson’s disease symptoms. Reduced volumes of bilateral sensorimotor and associative portions of the putamen and increased volumes of left inferior frontal and right inferior temporal regions were associated with increased freezing of gait episodes in 151 patients.^37^ Reduced volume of the nucleus basalis of Meynert was associated with worsening of gait measures, including variability of swing and stride timing, in 22 patients at a shorter-term follow-up.^57^ In a subset of 11 patients, swing time variability was associated with reduced nucleus basalis of Meynert volume at a three-year follow-up.

Non-voxel-based morphometric analyses have been applied in patients with Parkinson’s disease to assess volumetric measures in relation to DBS motor outcomes. In semi-automated approaches, one study identified no association of lateral ventricular volumes in 37 patients with idiopathic Parkinson’s disease on 4-month motor symptom improvement.^48^ In contrast, decreases in ventricular volume markers and linear ratios, namely bicaudate ratio, Evans’ index and third ventricular width, were reported in patients with unsatisfactory 6-month UPDRS III outcomes relative to those with satisfactory UPDRS III outcomes.^34^

Increases in FreeSurfer-derived volumes of the lateral and third ventricles and reduced volumes of the thalamus were significantly associated with lower UPDRS III outcomes up to a 15-month period in 86 patients with idiopathic Parkinson’s disease.^45^ Pre-operative left and right subthalamic nucleus (STN) volumes, used as the implantation target for DBS, were not correlated with UPDRS III outcomes 6-month following implantation in 59 patients with idiopathic Parkinson’s disease.^58^ Logistic regression outlined right thalamus and bilateral anterior cingulate volumes to be increased in a patient group with satisfactory, relative to a group with unsatisfactory UPDRS III outcomes.^58^ Additionally, correlations outlined bilateral anterior cingulate volume to be positively associated, whilst left precentral and right postcentral areas were negatively associated with post-operative UPDRS III outcomes.

Aside from regional volume, cortical thickness is a marker of gross morphology which measures the distance between the pial surface of grey matter and the grey matter-white matter border.^89^ In a sample of 31 patients with idiopathic Parkinson’s disease, there was found to be correlations between the reduced cortical thickness of right paracentral and left and right superior frontal clusters with poorer UPDRS III motor outcomes.^46^ Furthermore, patients with increased cortical thickness in the left and right superior frontal and left precuneus, superior temporal, inferior parietal and superior parietal clusters required lower stimulation voltages for improved outcomes. Other studies reported associations of increased cortical thickness of the left lateral occipital cortex,^31^ middle and superior frontal,^61^ and right inferior precentral^23,24,29,32,38,39,41,50,52,54‐56^ areas with greater UPDRS III motor outcomes. Specific to poorer gait symptom severity, associations were observed for the reduced cortical thickness of left postcentral regions, bilateral premotor and left inferior and superior parietal cortex.

### Structural connectivity analyses

**Figure 6:**
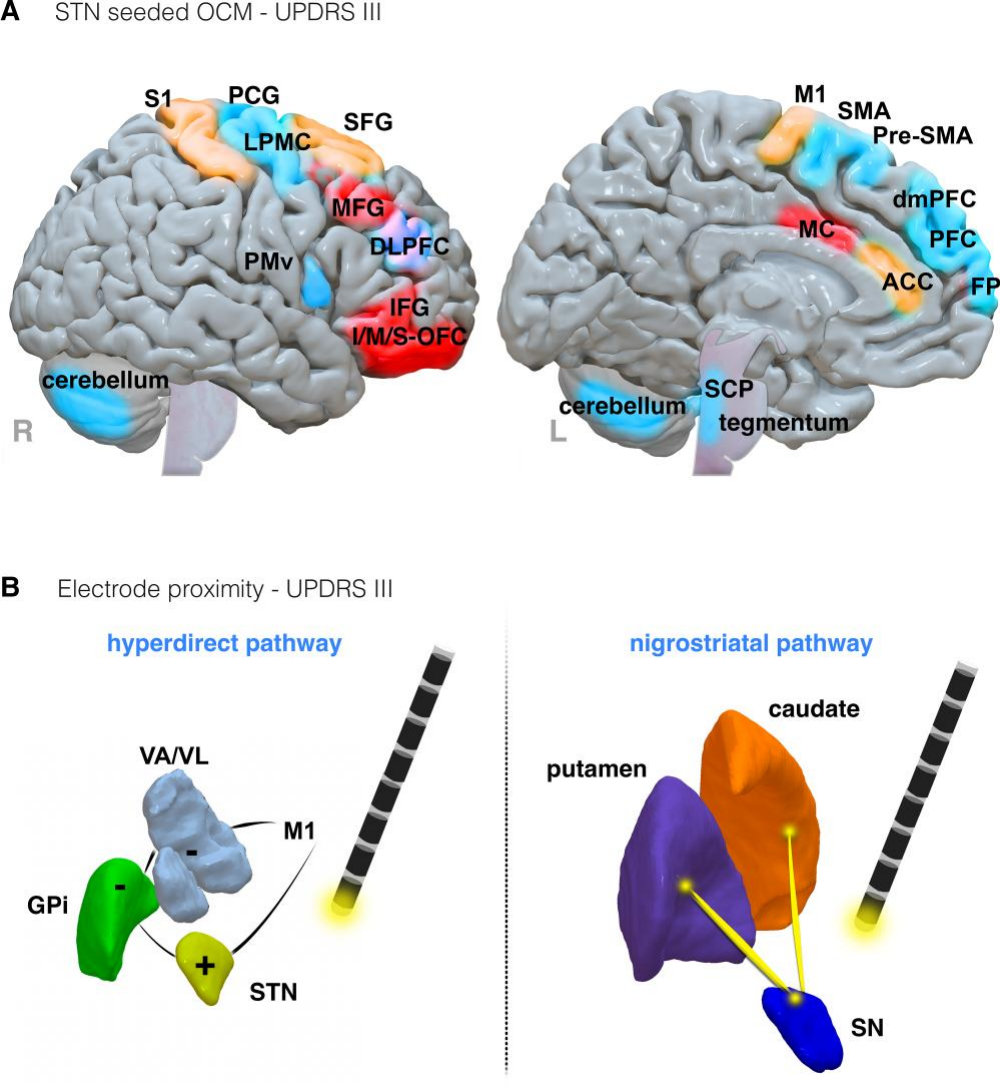
**Results summary of diffusion results from Parkinson’s disease studies.** (**A**) Brain regions indicated in UPDRS III motor outcomes, seeded from the STN, and in one study the STN and caudal ZI. (**B**) Basal ganglia pathways implicated with motor symptom improvements following closer EP. Left: hyperdirect pathway (reflects the primary motor cortex connection to the STN, with excitation (+) towards the GPi, and inhibition (−) of the motor thalamic nuclei and primary motor cortex (M1)). Right: Nigrostriatal pathway (reflects the connections from the SN to the dorsal striatum (caudate and putamen). Regions and tracts implicated in symptom improvements, worsening or both are coloured in blue, red, and orange, respectively. Abbreviations: ACC, anterior cingulate cortex; DLPFC, dorsolateral prefrontal cortex; dmPFC, dorsomedial prefrontal cortex; FP, frontal pole; IFG, inferior frontal gyrus; I-OFC, inferior orbitofrontal cortex; LPMC, lateral premotor cortex; MC, middle cingulate; MFG, middle frontal gyrus; M-OFC, middle orbitofrontal cortex; PFC, prefrontal cortex; PMC, premotor cortex; premotor ventral, PMv; S1, primary somatosensory cortex; SCP, superior cerebellar peduncle; SFG, superior frontal gyrus; S-OFC, superior orbitofrontal cortex; SMA, supplementary motor area; VA, ventral anterior nucleus; VIM, ventral intermediate nucleus; VL, ventral lateral nucleus

**Figure 7:**
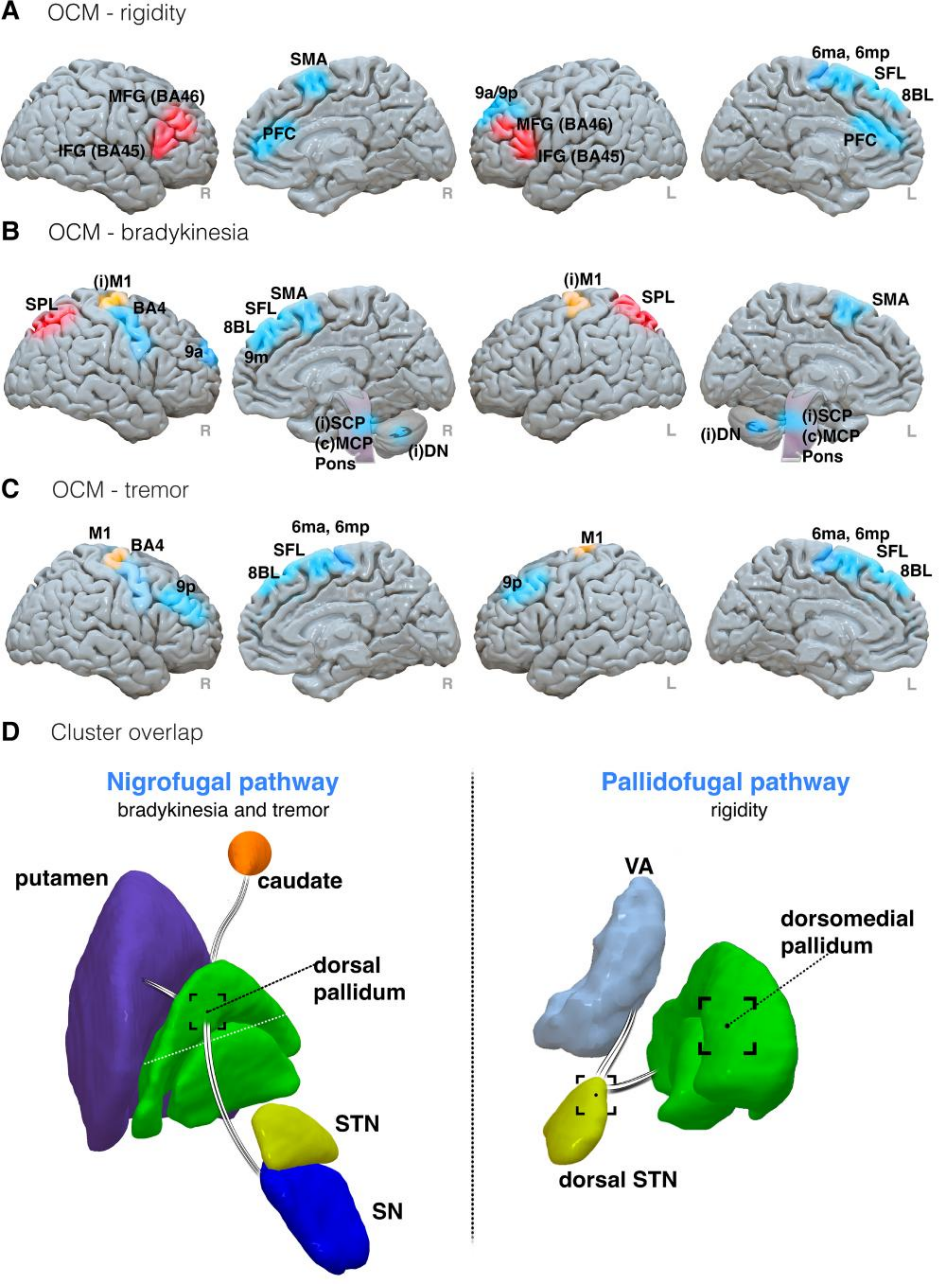
**Results summary of symptom-specific diffusion results from Parkinson’s disease studies.** Symptom-specific improvements associated with rigidity (**A**), bradykinesia (**B**) and tremor (**C**) improvements through outcome connectivity mapping. Regions associated with improvement, worsening or both are coloured blue, red and orange, respectively. (**D**) Clusters for improvement associated with tract overlap. Left: the nigrofugal pathway originates at the SN, where it intersects the dorsal pallidum, reaching the caudate and putamen. Right: the pallidofugal pathway originates at the dorsomedial pallidum where it intersects the dorsal STN with onward projections to thalamic motor nuclei. Abbreviations: BA, Brodmann area; (**C**) MCP, contralateral middle cerebellar peduncle; IFG, inferior frontal gyrus; (**I**)M1, ipsilateral primary motor cortex; (**I**)SCP, ipsilateral superior cerebellar peduncle; MFG, middle frontal gyrus; PFC, prefrontal cortex; M1, primary motor cortex; SFL, superior frontal language area; SPL, superior parietal lobe; SMA, supplementary motor area; VA, ventral anterior nucleus

A total of 20 studies investigated dMRI data from patients with Parkinson’s disease (summarized results are collectively presented in Figs. 6 and 7).

Most studies undertook an OCM approach, with 12 studies exploring contact profiles from seeds at the STN,^23,24,29,32,38,39,41,50,52,54‐56^ and one study assessing STN or caudal zona incerta (ZI).^35^ Other studies seeded from the pedunculopontine nucleus (PPN) in a sample of patients with postural instability,^49^ the globus pallidus internus (GPi) for assessing camptocormia symptoms,^40^ and also from patient-specific implantation regions associated with stimulation-induced or non-stimulation-induced dyskinesia (SID) symptoms.^53^

Due to the large number of studies assessing the relationship between regions associated with contact efficacy across various motor symptoms in Parkinson’s disease, significantly identified structurally connected regions are briefly summarized in Table 3 and Figs. 6 and 7 (OCM summaries for paraesthesia and motor contractions, SID and camptocormia outcomes are presented in Supplementary Fig. 1).

Two studies investigated diffusion tensor metrics in association with UPDRS III motor outcomes. In one study, fractional anisotropy, reflecting the degree of orientation, was used to define the connectivity of streamlines from STN to an atlas parcellation in patients with idiopathic Parkinson’s disease.^42^ Using a classifier to identify efficacious contacts, patients with optimal outcomes showed greater connectivity, with the thalamus, hippocampus, pallidum, primary motor cortex, supplementary motor area (SMA) and SFG. These connectivity features could be used to predict contact efficacy in a validation cohort (split into 58 patients for testing and 19 for validation) with 85% accuracy. In another study, increased axial and radial diffusion metrics extrapolated from streamlined connectivity between the basal ganglia and frontal/motor regions were associated with poorer UPDRS III motor outcomes.^32^

Network characteristics derived from graph theory were applied to whole-brain, atlas-based tractography in one study.^28^ In a cohort of 15 patients, greater eccentricity, theorized to reflect a measure of long-range regional integration, in left frontal and central modules was associated with increased UPDRS III motor outcomes. Receiver operating curves identified left medial and dorsolateral superior frontal gyri and inferior frontal gyri in the left frontal module and central areas including the left SMA and cingulate gyri to be greatest associated with motor outcomes.

The importance of electrode location relative to specific white matter tracts was also investigated. Closer electrode proximity (EP) was associated with greater clinical motor outcomes in relation to the hyperdirect pathway (Fig. 6B, left),^28^ the dentatorubrothalamic tract (DRTT)^60^ and left STN to the ipsilateral nigrostriatal pathway (Fig. 6B, right).^60^ Importantly, both deterministic and probabilistic approaches showed equivalency in the reconstruction of the nigrostriatal pathway, indicating robustness across tractography approaches. In one study, a trend towards significance for DRTT proximity was observed in nine patients.^51^

Symptom-specific improvements have been associated with tract-specific overlap of VTA clusters.^25^ Rigidity improvements were associated with overlap of pallidofugal pathways (Fig. 7D, left) whilst tremor and bradykinesia improvements were associated with overlap of nigrofugal pathways (Fig. 7D, right).

## Dystonia syndromes

### Morphometric findings

**Figure 8:**
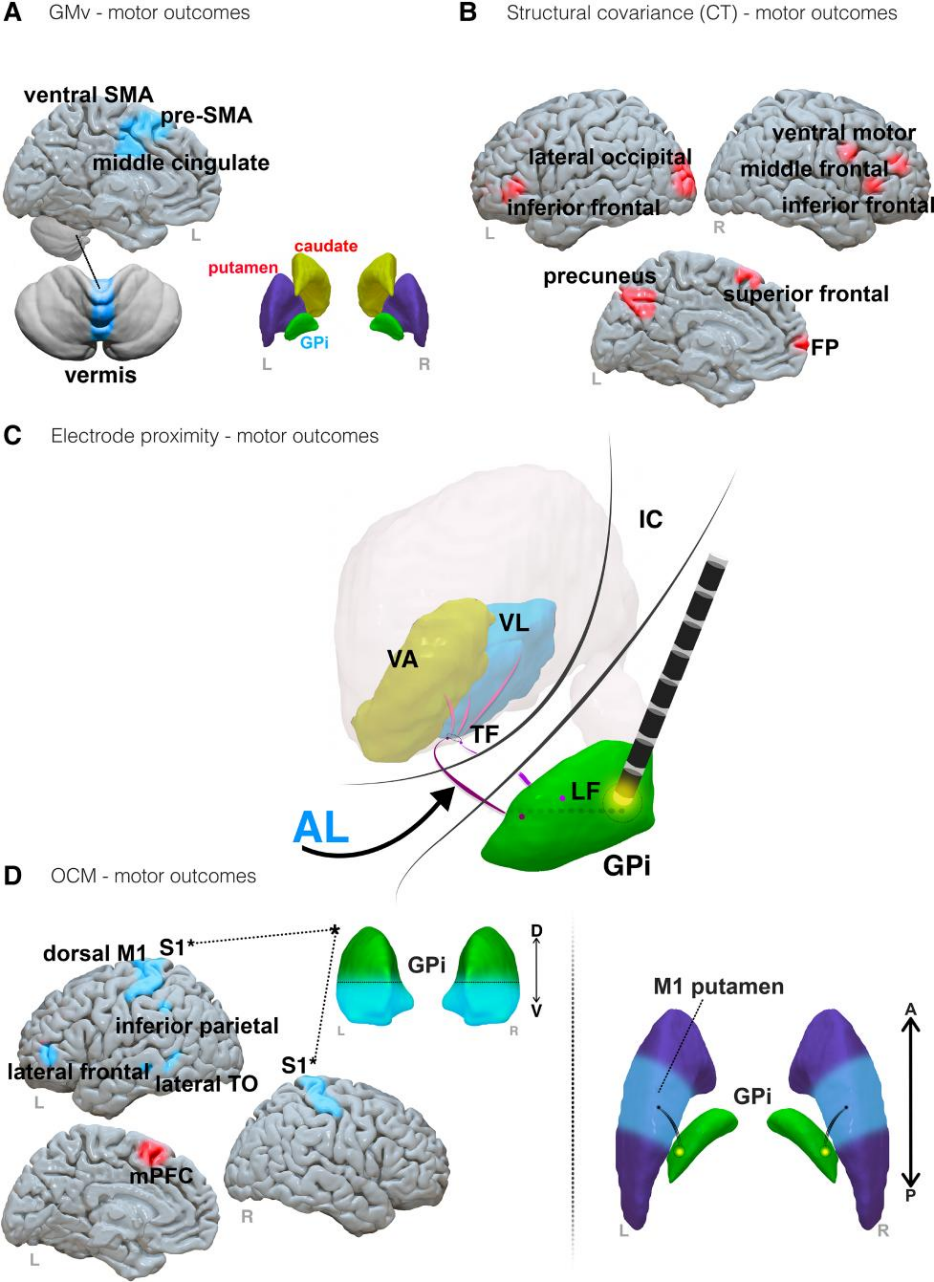
**Results summary from dystonia syndrome studies.** (**A**) Cortical, subcortical and cerebellum brain regions associated with grey matter volume (GMv) changes and dystonic motor symptom improvements. (**B**) Brain regions identified through structural covariance networks using cortical thickness (CT) and dystonic motor symptom worsening. **(C)** The AL pathway (blue) identified in association to closer EP for positive motor outcomes. (**D**) Left: brain regions associated with symptom changes through OCM. *Ventral GPi seeding and connectivity with S1 associated with symptom improvements. Right: pallido-putaminal (middle, M1 putamen) connectivity. Note, regions associated with positive and negative motor outcomes are presented in blue and red, respectively. Abbreviations: FP, frontal pole; IC, internal capsule; LF, lenticular fasciculus; mPFC, medial prefrontal cortex; SMA, supplementary motor area; M1, primary motor cortex; S1, primary somatosensory cortex; TO, temporal occipital; TF, thalamic fasciculus; VA, ventral anterior nucleus; VL, ventral lateral nucleus

Six studies assessed morphometry in patients with dystonic syndromes (main findings are presented in Fig. 8).

One study performed manual image inspection on a cohort of patients with early onset isolated dystonia, identifying an association between an increased number of GPi hypointensities from T1-weighted images and GPi hyperintensities from T2-weighted images, and poorer BFMDRS outcomes.^64^

One voxel-based morphometry study was identified in this review. In 16 patients with X-linked dystonia-parkinsonism, an association of reduced caudate nucleus volume with poorer BFMDRS outcomes was identified.^62^ Increased GMv clusters in the middle cingulate (MC) gyrus, pre-SMA and cerebellar vermis in patients with generalized and cervical dystonia were reported in a group of patients with optimal BFMDRS and TWSTRS improvements, respectively, relative to sub-optimal responders.^63^ A correlational analysis identified a significant association of increased grey matter density in the left MC gyrus and ventral SMA clusters with the clinical pre-and-post-operative motor score change (Fig. 8A).

Semi-automated morphometric analyses were performed in two studies in patients with generalized dystonia. Following the demarcation of GPi boundaries, three-dimensional volumetric reconstructions were performed using in-house stereotactic modelling. Associations of increased right and bilateral GPi volumes with greater BFMDRS outcomes in cohorts of 30 and 40 patients with primary generalized dystonia, respectively, were reported.^70,71^

Morphometric analysis using FreeSurfer-derived structural covariance and graph theory was performed in one study (Fig. 8B).^65^ Using group-based cortical thickness structural covariance networks, widespread reductions including right ventral motor, inferior and middle frontal clusters, left superior frontal, precuneus and lateral occipital clusters, were observed in patients with generalized and cervical dystonia with sub-optimal responses on BMFDRS and TWSTRS clinical scales, respectively. The patient group with greater clinical improvements also required lower stimulation amplitudes at these regions to receive more beneficial outcomes. Patients with sub-optimal outcomes also showed smaller bilateral caudate and putamen volumes, relative to patients with satisfactory outcomes. The authors indicated path length and clustering coefficient metrics derived from the group-based covariance networks to infer greater global dysconnectivity in patients with moderate outcomes. Specific to central and frontoparietal regions, increased centrality and clustering metrics were obtained, indicated by authors to reflect increased network segregation and organization inefficiency.

#### Tractography findings

A total of four studies used tractography to model structural connectivity in patients with dystonia syndromes. Tract overlap was performed in one study whereby contact proximity to the ansa lenticularis (AL) was significantly correlated with BFMDRS improvement in 10 patients with focal and segmental dystonia (Fig. 8C).^69^

Three studies applied OCM (Fig. 8D). Dorsal and ventral GPi implant connectivity patterns were compared in one study with patients with focal or segmental dystonia.^68^ Whilst both implantation areas offered sufficient motor symptom reduction, ventral profiles achieved greater efficacy and were associated with greater connectivity to the left and right primary sensory cortex. Dorsal profiles were associated with a stronger connectivity to the left and right premotor cortex (PMC) and pre-SMA. In 39 patients with generalized dystonia, stronger connectivity to the left dorsal primary motor cortex, lateral frontal, inferior parietal, and lateral temporo-occipital regions were associated with positive UDRS outcomes.^66^ Negative UDRS outcomes were associated strongest with connectivity to the left medial PFC. In 19 patients with cervical dystonia, greater connectivity to the putamen was associated with positive motor outcomes.^67^ Parcellation of the putamina outlined greater pallidal connectivity to a middle (primary motor cortex) area to be significantly correlated with greater motor outcomes.

### Essential tremor and other tremor disorders

#### Tractography findings

**Figure 9:**
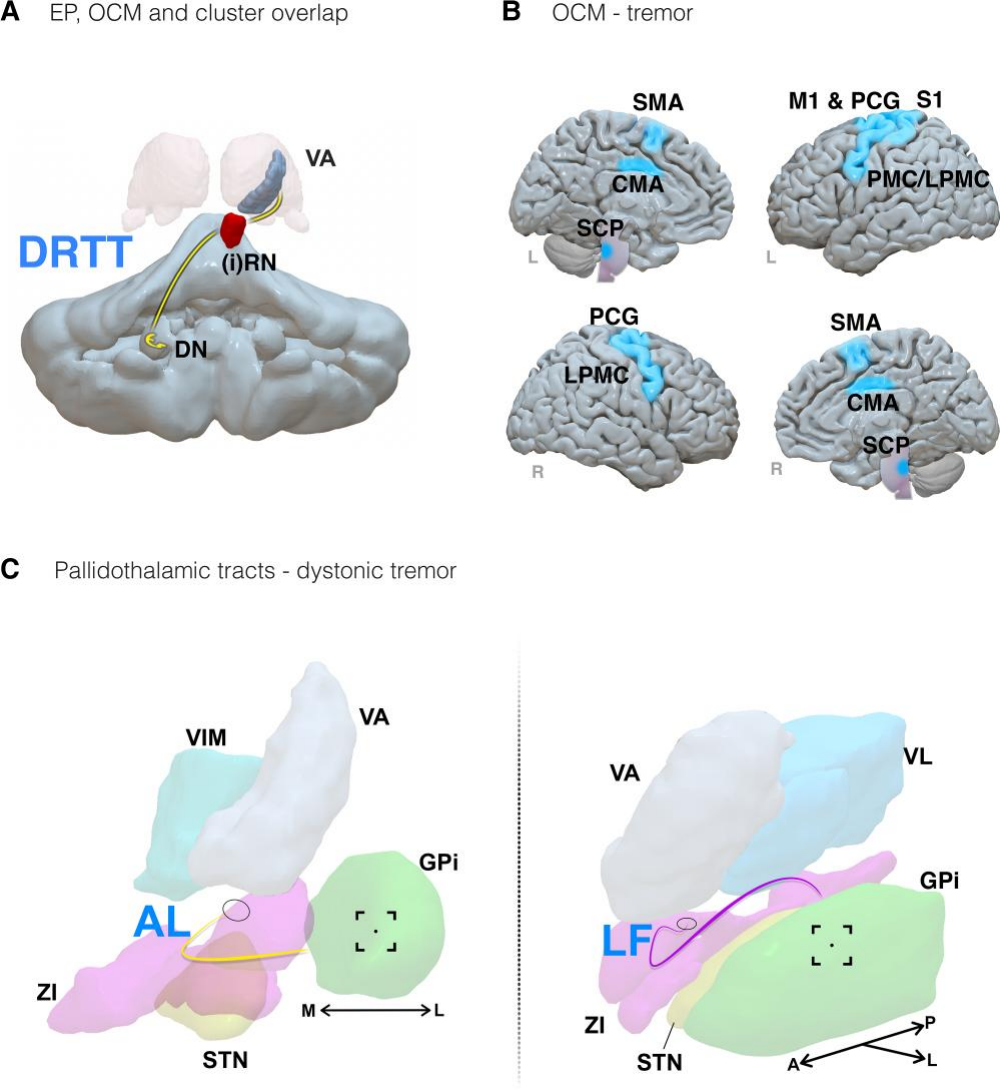
**Results summary of findings from tremor studies.** (**A**) The DRTT, outlined as efficacious for tremor improvement via EP, OCM and cluster overlap approaches. (**B**) Brain regions associated with tremor improvement through OCM. (**C**) A schematic of pallidothalamic tracts involved in dystonic tremor improvement. Left: the AL pathway [in yellow; viewed anteriorly with the medial (**M**) and lateral (**L**) axis included]. Right: the lenticular fasciculus (LF) pathway [in purple, viewed from a superior anterolateral perspective with the anterior (**A**), posterior (P) and lateral (**L**) axes included]. The circles at the end of each tract reflect the point of convergence for the thalamic fasciculus. Abbreviations: CMA, cingulate motor area; DN, dentate nucleus; GPi, globus pallidus internus; (**I**)RN, ipsilateral red nucleus; LPMC, lateral premotor cortex; M1, primary motor cortex; S1, primary somatosensory cortex; PCG, precentral gyrus; PMC, premotor cortex; STN, subthalamic nucleus; SCP, superior cerebellar peduncle; SMA, supplementary motor area; VA, ventral anterior nucleus; VIM, ventral intermediate nucleus; VL, ventral lateral nucleus; ZI, zona incerta

Identified tremor studies focused solely on the use of tractography-based analyses (results are summarized in Fig. 9).

Five studies investigated the DRTT in relation to clinical motor outcomes. In patients with essential tremor, two studies identified greater motor outcomes when electrode contacts were in closer proximity to the DRTT,^73,75^ although, in one study, no significant relationship was observed across five patients.^81^ In one study, greater overlap of VTA with the DRTT was observed to result in greater motor improvements.^79^ The effect of proximity to the DRTT was also observed in a small sample consisting of patients with essential tremor, tremor-dominant Parkinson’s disease, and myoclonic dystonic tremor.^74^

With regard to OCM, two studies seeded connectivity from the ventral intermediate nucleus of the thalamus (VIM). In a sample of 12 patients with either tremor-dominant Parkinson’s disease or essential tremor, connectivity to the precentral gyrus (PCG), supplementary and cingulate motor area (CMA), lateral premotor cortex (LPMC) and superior cerebellar peduncle (SCP) were associated with greater FTM improvements.^77^ Additional efficacious subcortical connectivity included the ipsilateral pallidum and brainstem. Ineffective contacts were identified to activate the same network of regions, shifted anteriorly. In another study, effective tremor suppression occurred when contacts were connected to the PCG and cerebellum and brainstem regions in patients with essential tremor.^80^ Seeding from the VIM and ZI, connectivity to a tract between the primary motor cortex and the cerebellum, intersecting the motor thalamus, was associated with greater clinical tremor improvement.^72^

Two studies seeded connectivity from contacts at the VIM/ventral oral posterior border of the thalamus and tested the predictive ability of normative connectomes using cross-validation. Structural connectivity to primary, supplementary and premotor cortices, sensory cortex and a tract corresponding to the DRTT were outlined as significant predictors for contralateral essential tremor improvements.^78^ This result was obtained by training on a cohort of 83 patients and validated on 14 patients. In a cohort 20 patients with essential tremor and 20 patients with dystonic tremor, the role of decussating and non-decussating DRTTs were explored.^82^ Leave-one-out cross-validations using group tract maps weighted by contralateral tremor improvement identified the pattern of the DRTT (combined non-decussating and decussating fibres) as predictive of outcomes for patients with essential tremor, and the pallidothalamic tract as predictive of outcomes for patients with dystonic tremor.

## Discussion

In this review, 60 studies identified structural features from MRI that could possibly account for and predict motor outcomes from DBS in patients with Parkinson’s disease, dystonia syndromes and tremor. Given the large number of studies identified in this review, structural features appear to be a promising approach. Studies highlighted a variety of morphometric and connectivity-based measures across all movement disorders, along with iron-based features applied specifically to patients with Parkinson’s disease. Importantly, the diversity of reported markers and techniques highlights the lack of unified understanding of aetiologies and exemplifies the clinical heterogeneity of pathophysiological mechanisms across disorders.

### Deep brain stimulation on systems for motor control

Viewing motor disorders as aberrations to widespread networks is now recognized as being paramount to enhance our understanding of disorder aetiology and maximize therapeutic benefits received from DBS. Although the basal ganglia have been central to our understanding,^111‐113^ research has identified complex interfacing across whole-brain networks to give rise to motor control.^114,115^ Alterations to regions or connections within systems have been shown to result in disease or disorder.^116^ Consequently, our understanding of DBS effects extending the local site of stimulation corroborates a systems-level approach to network neuromodulation, by therapeutically disrupting the underlying pathological circuitry of a given movement disorder.^117‐120^ Indeed, the change in view of diagnostic constructs is nicely summarized by the suggestion of a nomenclature change of DBS to ‘electrical neuro-network modulation’ which better reflects DBS mechanistically and how movement disorders (and perhaps most disorders and diseases) should be treated.^121^

The interaction between neuromodulation and brain structure is unclear. Given that neuromodulatory effects have been shown to be sensitive to changes in brain state,^122,123^ it would make sense that the structural architecture of the brain—in terms of grey and white matter integrity—would instantiate a mediating factor for therapeutic efficacy. Interesting work has indicated that both structural grey matter volumetric changes and white matter changes can occur following neuromodulation from DBS.^124‐127^ However, it remains unclear whether these findings are a result of the intervention itself, or pathological processes related to circuitry change within the disorder.

### Parkinson’s disease: more than the basal ganglia

Relative to other movement disorders, the neuropathology of Parkinson’s disease is well-established.^128^ The theoretical models of basal ganglia functioning have formed the basis for our understanding of motor control.^129,130^ Classically defined ‘direct’, ‘indirect’ and ‘hyperdirect’ basal ganglia pathways have been popularly applied as physiological mechanisms for control of the basal ganglia-thalamo-cortical loop, with prominence for Parkinson’s disease,^131^ aiding our understanding of circuitry dysfunction at a systems level.^132^

Neurodegeneration of the substantia nigra (SN) pars compacta is a main hallmark of Parkinson’s disease presence and progression,^133^ showing susceptibility to enhanced localized iron deposition^134^ and dopaminergic neuronal loss post-mortem.^135^ The positive associations of increased iron-related presence for Parkinson’s disease in the STN and SN indicate a role of dopaminergic pathology in the mediation of DBS efficacy.^43,44^ Interestingly, STN GMv was not associated with DBS outcomes but was reduced relative to healthy controls,^45^ a finding that has also been observed at 7 Tesla.^136^ Volumetric decreases of the putamen,^37^ thalamus^58,59^ and ventricular enlargements,^34^ may perhaps better reflect progressive disease changes which associate more closely with DBS efficacy. In contrast, STN volumes may reflect a more general marker of the disease’s presence. Animal models suggest that dopaminergic midbrain neuronal loss results in marked reductions of STN efferent connections in the hyperdirect pathway, and synaptic changes with the pallidum.^137,138^ However, given that Parkinson’s disease is a neurodegenerative disorder and dopaminergic neuronal loss continues through the late stages of progression,^139^ it remains unclear as to why volumetry of the STN may not be concordant with changes in motor symptoms in the disease.

White matter tract importance for DBS outcomes in Parkinson’s disease was evident in the present review. Closer contact proximity and increased overlap of pallido- and nigrofugal basal ganglia pathways appeared to be beneficial for improving clinical motor outcomes following DBS.^25,28,60^ As mentioned, tract importance underpins the necessity to focus on extending outside the local stimulation area. Corroborative findings for structural variability and connectivity from contacts deemed as efficacious in cerebellar and widespread cortical regions, particularly frontal and sensorimotor, highlight the whole-brain network aspect for determining DBS response.^23,24,29,31,32,35‐42,46,49,50,52‐56,58^

Dysfunctional neurotransmission of dopamine has dominated as the primary pathophysiological explanation for Parkinson’s disease symptoms, given its fundamental modulatory role within the cortico–basal–ganglia architecture.^140,141^ Importantly, regions constituting non-dopaminergic systems were pathologically highlighted in the current review. In particular, the PPN and nucleus basalis of Meynert constitute centres for large cholinergic afference and are regions that have emerged as susceptible in patients with Parkinson’s disease.^142‐144^ Importantly, cholinergic neurons are also present in the striatum and have been shown to regulate medium spiny neurons of direct and indirect basal ganglia pathways,^145^ and regulate dopaminergic release in the striatum.^146^ The cholinergic system has been identified to play a key role in mobility functioning with alterations resulting in postural instability, gait issues and falling in patients with Parkinson’s disease.^147,148^ Here, we observed supporting evidence of PPN and nucleus basalis of Meynert structural variability as potential structural correlates for axial symptom response in patients with bradykinesia and predominant posture and gait issues.^49,50,57^ Whilst the mechanisms for how cholinergic systems are disrupted in patients with Parkinson’s disease requires further investigation, this review sheds light on the importance of recognizing the system-level cascade of disorder pathology and potential DBS targets for patients with axial-dominant symptoms.

### Sensorimotor importance in dystonic syndromes

Dystonia syndromes are highly prevalent movement disorders, but aetiologies are poorly understood and heterogeneous.^149‐151^ This is evident from the current review with studies identifying patients with focal (including cervical), segmental and generalized dystonia’s along with clinical phenotypes such as X-linked dystonia-parkinsonism.

Unlike Parkinson’s disease, primary dystonia syndromes have no evidence of neurodegeneration and no hallmark brain-based pathologies present.^149^ However, the GPi of the basal ganglia are the most favourable nuclei for optimal therapeutic benefits, indicating analogous similarity in circuitry dysfunction.^152^ Electrophysiological GPi recordings support abnormal pallidal signalling within the thalamo-cortical system.^153,154^Amelioration of atypical functioning may be achieved by GPi-DBS resulting in therapeutic dystonic relief. Compared with the observation that STN volumes were not associated with DBS outcomes for Parkinson’s disease,^45^ GPi volumes were associated with importance for motor outcomes in primary dystonia.^70,71^ The reasons for this distinction may be due to several factors. First, the manual delineation technique used for GPi reconstruction may offer a more accurate estimation of volumetry. Automatic approaches may be less sensitive to quantifying small subcortical nuclei, especially given that T1-weighted MRI were used, which provides poor contrast for boundary estimations of the STN. Second, the therapeutic benefits obtained from patients with dystonia syndromes are not instantaneous in comparison to Parkinson’s disease, indicating more gradual neuroplastic changes underpin neuromodulatory effects.^1,155^ Pallidal volumes may, therefore, play a more prominent role in promoting longer-term plastic changes, especially given that they comprise the primary output nodes of the basal ganglia.

Other identified basal ganglia nuclei included the caudate and putamen, making up the dorsal striatum, from which reduced volumes and signal abnormalities were indicative of poorer DBS response.^62,64,65^ The dorsal striatum is a key module for basal ganglia input and plays a major role in action selection.^156^ The dorsal striatum appears to be particularly susceptible in genetic dystonic syndromes with large volumetric reductions evident in late-stage X-linked dystonia-parkinsonism.^157^ Despite initial evidence indicating the caudate to primarily be involved in cognition and the putamen in motor functions, extensive and widespread prefrontal and sensorimotor cortico-striatal connectivity and unclear anatomical boundaries indicate functional integration of regions.^158^ Further evidence suggests that the caudate nucleus is susceptible to genomic-based changes in Huntington’s disease,^159^ and microstructural changes in cervical dystonia.^160^ Striatal dysfunction is regarded as a key mediator of abnormal cortical plasticity that is associated with dystonia syndromes.^161,162^ Modulation of this abnormal plasticity has been proposed to underpin the therapeutic effects of DBS, highlighting the area as key to mediating clinical outcomes.^163^

The association of basal ganglia regions with cerebellar and sensorimotor cortical activity is the primary theory underpinning dystonia pathophysiology. Abnormalities of sensory processing,^164^ and synchronization and overactivation of sensorimotor networks are well-defined features of dystonia syndromes.^165^ Negative correlations between the somatosensory cortex and cerebellum, derived from functional MRI, have been associated with favourable outcomes in dystonia and present as a signature of circuitry dysfunction.^166,167^ In this review, pallidum to putamen-primary motor cortex connectivity as a predictor for outcomes in patients with isolated cervical dystonia is in line with the abnormal motor component of dystonia.^67,168^ DBS has been indicated to normalize oscillatory coherence with and decrease the overactivity of the primary motor cortex.^169,170^ Furthermore, identified optimal outcomes arising from ventral GPi implantations with association to increased connectivity with primary somatosensory cortex provide additional support for sensorimotor networks as a prominent marker for neuromodulatory efficacy.^68^

Finally, although indicated as a symptom of Parkinson’s disease, efficacious contacts determined by GPi-primary somatosensory cortex and STN-pre/supplementary motor cortex connectivity in patients with camptocormia highlights the salience of sensorimotor network involvement for abnormal posturing symptoms across disorders.^40,41^ Further exploration of these circuits may help to elucidate a therapeutic target aimed at altering sensorimotor networks to control dystonic and postural symptoms.

### Cerebellar involvement in tremor networks and beyond

Despite essential tremor being the most prevalent movement disorder worldwide, the aetiology (including other tremor subtypes) is not well understood. Essential tremor manifests as a kinetic tremor (4–12 Hz) that can affect all parts of the body.^171^ Dystonic tremor is typically diagnosed with postural abnormalities and irregular tremor amplitude and frequency.^172^ Parkinsonian tremor typically affects the upper extremities and can occur as either action tremor or rest tremor (4–6 Hz).^173^

In this review, cerebellar involvement in clinical outcome mediation was identified across multiple structural MRI domains and tremor disorders. Initial pathomechanisms for essential tremor implied the inferior olivary nucleus as a centre for tremerogenesis.^174^ However, this theory has fallen out of favour with a lack of evidence for both activation using positron emission tomography,^175^ and post-mortem structural change in patients with essential tremor.^176^ Instead, evidence supports essential tremor as a neurodegenerative disorder of the cerebellum due to observations of cellular neurodegeneration in post-mortem examinations.^177^

The cerebellum plays a crucial role in calculating and predicting movements based on error in the form of large-scale sensorimotor feedback.^178,179^ Dysfunction of cortico-cerebellar circuitry has been proposed to result in excessive oscillatory feedback and the consequent tremor generation.^180,181^ The neuromodulation of a ‘tremor network’ through cerebellar circuitry has been proposed to disrupt aberrant oscillatory activity, inducing corrective functioning and alleviating tremor activity.^76^ Two cerebellar tracts that have been heavily implicated in tremor pathophysiology are ‘short’ and ‘long’ loops. The former is said to reflect a short-range circuit comprising the dentate nucleus (DN) of the cerebellum and the inferior olivary nucleus, constituting a dentato-olivio-cerebellar pathway. The long loop pertains to projections from the DN towards the thalamus for cortico-cerebellar connectivity, likened to the anatomical course of the DRTT.^180^

In the present review, dMRI outlined compelling evidence for DRTT involvement in tremor suppression across multiple tremor pathologies. Importantly, this was the case not only when the DRTT was explicitly investigated,^74‐76,79^ but also in whole-brain exploratory approaches.^72,78,80,82^ Whilst the study identifying no association of contact proximity to the DRTT may have been restricted by a small sample size,^81^ deterministic tractography used in the study has outlined a poorer ability to reconstruct the DRTT, relative to probabilistic tracking.^182,183^ Importantly, tractography performance is dependent upon the tract(s) of interest, and the purpose for reconstruction such that probabilistic tractography does not always offer superior efficacy for tract reconstruction.^182,184,185^

Evidence for sensorimotor feedback being involved in tremor mechanisms was also indicated across studies. Connectivity with the cerebellum, brainstem and sensorimotor cortices was indicative of greater essential tremor improvement.^77,78,80^ Importantly, the role of motor regions indicated in Parkinson’s disease showed contrasting results across studies. Motor region connectivity proved beneficial in tremor-dominant Parkinson’s disease and tremor subscales of the UPDRS III,^24,39,80^ but also associated with poor contact efficacy on the UPDRS III tremor component in another study.^50^ The divergence in results here raises an important point regarding clinical heterogeneity, disorder understanding and therapeutic targeting that is discussed in the following section.

### Clinical heterogeneity and symptom-specific targeting

Movement disorders are rarely monosymptomatic and will often present with myriad symptoms, reflective of widespread clinical heterogeneity.^151,186,187^ Understanding the contribution of each brain region presents an incredibly complex task and requires patient cohorts that are as homogenous as possible to derive clinically meaningful inferences.

As mentioned, we observed many regions associated with positive outcomes to be associated with negative outcomes in other studies. Furthermore, networks pertaining to a specific symptom of a disorder likely constitute different white matter pathways and brain regions. For example, rigidity and bradykinesia likely constitute different circuitry pathologies which may not be optimally therapeutically targeted under conventional STN DBS. A lack of focality on specific network targeting is exemplified by findings that DBS targeting of STN and GPi appear to modulate extremely similar networks in patients with Parkinson’s disease.^188^ Identifying and targeting symptom-specific networks are the next steps for individualizing DBS treatment to optimize therapeutic benefits at an individual level.^189^

### Association versus prediction: an issue for finding generalisability

An important implication present in this review is the utility of markers that are inferred through means of association and correlation, versus prediction. Although many articles explicitly outlined the ‘predictive’ ability of identified structural marker(s), analyses were restricted to correlations/associations.^24‐27,30,33,37,38,43,44,46,48,57,59,62,65‐67,70,79,80^ In such cases, derived markers cannot be strictly predictive as they reflect a potential disease/disorder correlate within the sample to which the model was fit.

Validation of these markers through the means of testing on unseen (independent) data reflects a more robust method to infer finding generalisability.^190^ Leave-one-out cross-validation reflects a prediction of a single case (i.e. patient) by training a statistical model on the rest of the data and repeating this iteratively until all cases have been modelled.^191^ Studies utilizing this approach enable greater finding generalisability than associations constructed from the entire data set only, ^53,72,78,82^ by increasing chances of avoiding overfitting. However, even in leave-one-out cross-validation, the model is still only internally validated in the group.^192^ Studies applying external validation, through training and testing statistical models on independent data sets, offer perhaps the greatest representation of prediction. This was observed for single-centre splitting,^29,39,42,61^ and cross-centre testing and training sets.^56,78,193^ Cross-centre validation remains one of the best ways to construct generalizable, prognostic models.

### What is a good ‘response’ from deep brain stimulation?

Determining what is meant by ‘response’ to DBS is a subjective matter, complicated by symptomatic differences, surgical factors, disorder severity, clinical follow-up and patient expectations. Additionally, smaller improvements (or declines) in symptoms that are subjectively more severe may not translate effectively when quantifying pre-and-post score changes, raising concern over the sensitivity towards patient-centred improvement.

In the present review, we identified a range of grouping thresholds and follow-up points used to distinguish patients with higher versus lower motor improvements. Determining which grouping threshold to use to dichotomize patients, and how to calculate the change are incredibly important aspects that will impact the inferences and conclusions derived from a study. Arriving at standardized notions for response level for treatment outcomes is an area that requires work but will massively benefit the quality of research.

### Patient-specific connectivity and normative connectomes

The number of studies identified in this review using dMRI outlines the interest in assessing aspects of white matter connectivity as biomarkers for clinical outcomes from DBS. In theory, patient-specific dMRI enables greater insight and inference for studies assessing DBS response given that it is reflective of the patient(s) in question. Furthermore, patient-specific dMRI is showing increasing clinical utility for optimizing intra-operative planning and targeting of DBS electrodes in relation to white matter fibres.^8,194,195^

As identified in the review, sample sizes of studies utilizing patient-specific dMRI tended to be small in comparison to studies utilizing normative connectomes. Whilst not patient-specific, normative connectomes do enable larger sample sizes of patients to be analysed in cases where dMRI data is not routinely acquired (i.e. in clinical care pathways) making it a time and cost-effective technique. In addition, pitfalls of poor test-retest reliability of individual dMRI data may be overcome by the greater signal-to-noise ratios offered by the larger sample sizes, and the specialized acquisition protocols that have been implemented during acquisition.^196‐198^

The question of to what extent statistical inferences may be improved by the use of disease-matched normative connectomes is undetermined as similar predictive abilities with healthy normative connectomes for post-operative outcomes have been observed.^56,199^

Whilst both applications will continue to improve our understanding of developing predictive biomarkers for DBS outcomes, the fundamental limitations of dMRI still hold for both. Inaccuracies of tract reconstruction in areas of crossing-fibres make the delineation of specific tracts an issue.^97,200^ Additionally, resolution of connection direction cannot be achieved, making efferent and afferent connectivity indistinguishable.^201^ Furthermore, the exact relationships between diffusion-derived metrics and white matter microstructure remain elusive.^202,203^

## Conclusion

Techniques focused on structural MRI assessment for post-operative motor outcome prediction offer great promise to identify patients more likely to benefit from DBS. In this review, we identified many applications to patient cohorts with Parkinson’s disease, dystonia syndromes and tremor. The integral role of cortical, subcortical, and cerebellar networks on the mediation of DBS response are observed across MRI methods and movement disorders. The variety of techniques identified in this review demonstrates the possibilities on the horizon for structural features from MRI. Differences of cortical thickness and brain volumes present corroborative markers that may be useful to inform candidate selection. Advancements of dMRI approaches to probe white matter tracts are helping to extend our understanding of disorder pathology and to provide alternative therapeutic targets. Future applications pose great promise to aid prediction of outcomes at the individualized level, and to help elucidate mechanisms that underly disorders.

## Supplementary Material

fcad171_Supplementary_Data

## Data Availability

Data sharing is not applicable to this article as no new data were created or analysed in this study.

## Abbreviations

ACC =: anterior cingulate cortex
AL =: ansa lenticularis
BA =: Brodmann area
BFMDRS =: Burke–Fahn–Marsden dystonia rating scale
BIC =: brachium of the inferior colliculus
CMA =: cingulate motor area
cMCP =: contralateral middle cerebellar peduncle
CT =: cortical thickness
cZI =: caudal zona incerta
DBS =: deep brain stimulation
DLPFC =: dorsolateral prefrontal cortex
dmPFC =: dorsomedial prefrontal cortex
dMRI =: diffusion MRI
DN =: dentate nucleus
DRTT =: dentatorubrothalamic tract
DTI =: diffusion tensor imaging
FAL =: anterolateral fasciculus
FoG =: freezing of gait
FoGQ =: freezing of gait questionnaire
FP =: frontal pole
FSL =: FMRIB Software Library
FTM =: Fahn–Tolosa marin clinical rating scale for tremor
GFQ =: gait and falls questionnaire
GMv =: grey matter volume
GPe =: globus pallidus internus
GPi =: globus pallidus internus
HCP =: human connectome project
IC =: internal capsule
IFC =: inferior frontal cortex
IFG =: inferior frontal gyrus
IML =: internal medullary lamina of the thalamus
iM1 =: ipsilateral primary motor cortex
I-OFC =: inferior orbitofrontal cortex
iPMC =: ipsilateral premotor cortex
iRN =: ipsilateral red nucleus
iSCP =: ipsilateral superior cerebellar peduncle
LF =: lenticular fasciculus
LPMC =: lateral premotor cortex
m-ALIC =: medial part of the anterior limb of the internal capsule
MC =: middle cingulate
MDS-UPDRS =: movement disorder society sponsored revision of the unified Parkinson’s disease rating scale
MFB =: medial forebrain bundle
M-FC =: middle frontal cortex
MFG =: middle frontal gyrus
MGH-USC =: Massachusetts General Hospital—University of Southern California
ML =: medial lemniscus
M-OFC =: middle orbitofrontal cortex
mPFC =: medial prefrontal cortex
M1 =: primary motor cortex
n =: sample size
nBM =: nucleus basalis of Meynert
NC =: normative connectome
OCM =: outcome connectivity mapping
OFC =: orbitofrontal cortex
PCG =: precentral gyrus
PFC =: prefrontal cortex
PMC =: premotor cortex
PMd =: premotor dorsal
PMv =: premotor ventral
PPMI =: Parkinson’s progression markers initiative
PPN =: pedunculopontine nucleus
PRISMA =: preferred reporting items for systematic reviews and meta-analyses
SCP =: superior cerebellar peduncle
SFC =: superior frontal cortex
SFG =: superior frontal gyrus
SFL =: superior frontal language area
SID =: stimulation-induced dyskinesia
SIP =: stepping-in-place task
SMA =: supplementary motor area
SN =: substantia nigra
S-OFC =: superior orbitofrontal cortex
SPL =: superior parietal lobe
STN =: subthalamic nucleus
S1 =: primary somatosensory cortex
TF =: thalamic fasciculus
TO =: temporal occipital
TWSTRS =: Toronto Western Spasmodic Torticollis rating scale
UDRS =: unified dystonia rating scale
UPDRS =: unified Parkinson’s disease rating scale
VA =: ventral anterior nucleus
VIM =: ventral intermediate nucleus
VL =: ventral lateral nucleus
VOa =: ventralis oralis anterior
VOp =: ventralis oralis posterior
VTA =: volume of tissue activation
ZI =: zona incerta
