# Supplementary material for: A structural magnetic resonance imaging review of clinical motor outcomes from deep brain stimulation in movement disorders
Source: Brain Commun. 2023 May 31;5(3):fcad171. doi: 10.1093/braincomms/fcad171 (PMC10257440; doi:10.1093/braincomms/fcad171)
Supplement: fcad171_Supplementary_Data [file fcad171_supplementary_data.pdf]

Supplementary methods

Database search strategies

PubMed search strategy

(Deep Brain Stimulation*[Title/Abstract] OR DBS[Title/Abstract] OR Stimulation[Title/Abstract] OR Modulation[Title/Abstract]) AND (Parkinson disease[Title/Abstract] OR Parkinson's disease[Title/Abstract] OR PD[Title/Abstract] OR dystonia[Title/Abstract] OR essential tremor[Title/Abstract] OR ET[Title/Abstract] OR tremor[Title/Abstract]) AND (Magnetic Resonance Imaging OR MRI OR Neuroimaging OR structural OR tractography OR diffusion tensor imaging OR DTI OR connectivity OR atrophy OR volume) AND (predict* OR outcome OR clinical OR biomarker OR preoperative)

Scopus search strategy

TITLE-ABS-KEY ({Deep Brain Stimulation} OR DBS OR stimulation OR modulation) AND TITLE-ABS-KEY (Parkinson* AND disease OR PD OR dystonia OR essential AND tremor) AND ({magnetic resonance imaging} OR MRI OR neuroimaging OR structural OR tractography OR {diffusion tensor imaging} OR DTI OR connectivity OR atrophy OR volume) AND (predict* OR outcome OR biomarker OR clinical OR preoperative)

Web of Science search strategy

AB=(Deep Brain Stimulation OR DBS OR Stimulation OR Modulation) AND AB = (Parkinson* Disease OR PD OR dystonia OR essential tremor OR ET) AND ALL=(Magnetic Resonance Imaging OR MRI OR Neuroimaging OR structural OR tractography OR connectivity OR diffusion tensor imaging OR DTI OR connectivity OR atrophy OR volume) AND ALL=(Predict* OR outcome OR clinical OR biomarker OR preoperative)

Supplementary Table 1 Systematic review data extraction summary

Authors	n[a]	DX	Target	MRI	Scale	Follow up[b]	Findings Summary
Parkinson's disease							
Bonneville et al, 2005[(26)]	40	PD	bilateral STN	semi-automated morphometry	UPDRS III	6 months	a trend of an association of reduced normalised mesencephalon surface volume with poorer outcomes
Hamasaki et al, 2010[(33)]	21	idiopathic PD	bilateral STN	VBM	UPDRS III	3 months	greater global white matter volume associated with greater outcomes
Price et al, 2011[(48)]	- 11<br>- 26<br>(37)	idiopathic PD	- unilateral GPi<br>- unilateral STN	semi-automated morphometry	UPDRS III	4 months	no significant association of lateral ventricular volume on outcomes
Sweet et al, 2014[(51)]	- 2<br>- 7<br>(9)	idiopathic PD	- unilateral STN<br>- bilateral STN	proximity (DT)	UPDRS III	undisclosed	a non-significant trend of closer contact proximity to DRTT being associated with greater outcomes
Lönnfors-Weitzel et al, 2016[(44)]	12	idiopathic PD	STN	T2 relaxation time	UPDRS III	12 – 26 weeks	reduced minimum STN T2 relaxation time associated with poorer outcomes
Vanegas-Arroyave et al, 2016[(54)]	22	idiopathic PD	bilateral STN	OCM (PT)	UPDRS III	3 and 6 months	effective contacts connected to the SFG and thalamus
Akram et al, 2017[(24)]	20	idiopathic PD	bilateral STN	OCM (PT)	UPDRS III	12 months	improvements in tremor, bradykinesia and rigidity associated with cortical motor and frontal connectivity
Horn et al, 2017[(35)]	- 51<br>- 44<br>(95)	PD	bilateral STN, cZI	OCM (NC)	UPDRS III	- 1 – 2 years<br>- 6 – 12 months	connectivity to frontal motor and cerebellum predicted outcomes in training and validation cohorts
Muthuraman et al, 2017[(46)]	31	idiopathic PD	bilateral STN	FreeSurfer	UPDRS III	≥ 3 months	increased frontal lobe cortical thickness associated with greater improvement and required DBS amplitude
Chen et al, 2018[(28)]	11	idiopathic PD	bilateral, unilateral STN	proximity (DT)	UPDRS III	1 month	closer contact proximity to "hyperdirect" pathway associated with greater outcomes
Koirala et al, 2018[(38)]	15	idiopathic PD	bilateral STN	graph theory (PT)	UPDRS III	3 months	network measures in cingulate, motor, and frontal cortex connectivity associated with outcomes
Karachi et al, 2019[(37)]	151	PD	bilateral STN	VBM	UPDRS III	1 year	reduced putamen volume and increased inferior gyri volume associated with increased freezing of gait

Krishna et al, 2019[39]	- 14<br>- 10<br>(24)	AR-PD	- bilateral STN -<br>unilateral STN	OCM (PT)	UPDRS III	1 year	predictive ability of symptom specific connectivity patterns to frontal and motor areas
Strotzer et al, 2019[50]	21	idiopathic PD	bilateral STN	OCM (PT)	UPDRS III	1 month	increased contact connectivity to ipsilateral SCP and ipsilateral DN associated with greater bradykinesia improvements
Younce et al, 2019[59]	86	idiopathic PD	bilateral STN	FreeSurfer	UPDRS III	≤ 15 months	increased lateral and third ventricle and reduced thalamus volumes in poorer responders
Avecillas-Chasin and Honey, 2020[25]	43	PD	bilateral STN	Overlap (NC)	UPDRS III	1 year	VTA cluster overlap with pallidofugal and nigrofugal pathways associated with outcomes
Erdogan et al, 2020[30]	39	PD	bilateral STN	manual inspection	UPDRS III	M: 1.5 weeks<br>R: 1 – 4 weeks	no significant association of lacunar lesions on motor outcomes
Frizon et al, 2020[31]	36	PD	bilateral STN	FreeSurfer	MDS-UPDRS III	6 months	reduced left lateral occipital cortical thickness associated with poorer motor outcomes
Hamed et al, 2020[34]	34	idiopathic PD	bilateral GPi	ZDA-PACS	UPDRS III	6 months	ventricular volume measures distinguished low and high responders
Lin et al, 2020[42]	77	idiopathic PD	bilateral STN	DWI metrics (DT)	UPDRS III	1 month	greater mean FA of connections to subcortical, motor, and frontal cortices thalamus associated with greater contact efficacy
Prent et al, 2020[47]	31	idiopathic PD	bilateral STN	proximity (DT)	UPDRS III	2 weeks	closer proximity of contact to DRTT associated with greater outcomes
Treu et al, 2020[52]	51	PD	bilateral STN	OCM (NC)	UPDRS III	R: 1 – 2 years	connectivity to cortical motor and sensorimotor regions associated with varying outcomes
Vassal et al, 2020[55]	9	idiopathic PD	bilateral STN	OCM (DT)	UPDRS III	6 months	effective contacts connected with brainstem, cerebellum, and cortical motor regions
Wilkins et al, 2020[57]	22	PD	bilateral sensorimotor STN	VBM	SIP and UPDRS III	M: 63.9 ± 31.9 d, 3 years	nbM volume reduction associated with swing time variability and stride time worsening
Yim et al, 2020[58]	81	PD	STN	NeuroQuant, VBM	UPDRS III	1 year	various subcortical and cortical region volume differences between greater and poorer responders
Abdulbaki et al, 2021[23]	36	PD	bilateral STN	proximity (PT)	UPDRS III	Median: 3 months<br>R: 1 – 7 months	closer contact proximity to DRTT associated with greater resting tremor improvement.

Cavallieri et al, 2021[27]	138	idiopathic PD	bilateral STN	manual inspection	MDS-UPDRS III	M: 8.4 – 6.26 years<br>R: 1 – 17 years	increased white matter hyperintensity presence associated with worse outcomes
Lai et al, 2021[40]	- 10<br>- 18<br>(28)	- camptocormia PD<br>- PD	bilateral GPi	OCM (NC)	MDS-UPDRS III	≤ 12 months<br>M: 7.3 ± 3.3 months	increased connectivity to right S1 associated with greater postural outcomes
Lai et al, 2021[41]	- 10<br>- 26<br>(36)	- camptocormia PD<br>- PD	bilateral STN	OCM (NC)	MDS-UPDRS III	≤ 12 months<br>M: 6.0 ± 2.2 months	increased connectivity to motor cortices associated with greater postural outcomes
Liu et al, 2021[43]	33	idiopathic PD	bilateral STN	QSM	MDS-UPDRS III	6 months	nigral iron accumulation features associated with motor and rigidity outcomes
Lu et al, 2021[45]	- 57<br>- 2<br>(59)	idiopathic PD	- bilateral STN<br>- bilateral GPi	FreeSurfer	UPDRS III	6 months	no associations of bilateral STN or intracranial volume on outcomes
Raghu et al, 2021[49]	8	PIGD-PD	bilateral PPN	OCM and DWI metrics (PT), FreeSurfer	GFQ and FOGQ	M: 15.38 ± 5.97 months<br>R: 12 – 29 months	connectivity patterns to PCG, S1 and SCP associated with outcomes. Increased parietal and premotor thickness associated with greater outcomes
Tsuboi et al, 2021[53]	- 8<br>- 6<br>(14)	PD	- bilateral pallidum<br>- unilateral pallidum	OCM (NC)	UPDRS III	M: 5.5 months<br>R: 4 – 9 months	different connectivity patterns from implantation sites associated with stimulation induced dyskinesia effects
Wang et al, 2021[56]	- 17<br>- 12<br>- 4<br>(33)	PD	bilateral STN	OCM (NC)	UPDRS III	- 1 year<br>- 6 months<br>- 2.5 – 7 months	effective contacts connected to cingulate, frontal and motor cortices
Chen et al, 2022[61]	94	PD	bilateral STN	FreeSurfer	MDS-UPDRS III	4 – 5 weeks	greater precentral inferior thickness associated with greater outcomes
Chen et al, 2022[29]	98	PD	bilateral STN	OCM (NC)	MDS-UPDRS III	4 – 5 weeks	widespread caudate, cingulate and cortical connectivity associated with outcomes
Gonzalez-Escamilla et al, 2022[32]	15	idiopathic PD	bilateral STN	FreeSurfer, DWI metrics and OCM (PT)	UPDRS III	3 months	connectivity, diffusion metrics and cortical thickness in motor and frontal cortical regions associated with outcomes

Jergas et al, 2022[36]	39	idiopathic PD	bilateral STN	VBM	UPDRS III	3 months	reduced frontoparietal volume associated with poorer outcomes
Li et al, 2022[60]	31	PD	bilateral STN	proximity (DT and PT)	MDS-UPDRS III	M: 12 ± 4 months	closer proximity from left STN to ipsilateral NSP in greater responders
Dystonia syndromes							
Gavarini et al, 2008[64]	25	dystonia (DYT1)	GPi	manual inspection	BFMDRS	2 years	poorer motor outcomes in patients presenting with more signal abnormalities in pallidum and putamen
Vasques et al, 2009[70]	40	primary GD	bilateral GPi	semi-automated morphometry	BFMDRS	M: 4.1 ± 1.9 years	greater right GPi volume with greater outcomes
Vasques et al, 2009[71]	30	primary GD	bilateral sensorimotor GPi	semi-automated morphometry	BFMDRS	M: 4.1 ± 1.98 years	greater bilateral GPi volumes associated with greater outcomes
Rozanski et al, 2014[68]	8	focal and segmental dystonia	bilateral GPi	OCM (PT)	BFMDRS	2 – 4 months	ventral GPi connectivity profiles associated with greater efficacy than dorsal GPi
Rozanski et al, 2017[69]	10	focal and segmental dystonia	bilateral GPi	proximity (PT)	BFMDRS	undisclosed	closer contact proximity to the ansa lenticularis associated with greater outcomes
Fečíková et al, 2018[63]	19	- GD<br>- CD	bilateral GPi	VBM	- BFMDRS - TWSTRS	M: 4 ± 2 years<br>R: 1 – 9 years	increased grey matter volume of pre-SMA, middle cingulate and cerebellar vermis in greater responders
Brüggemann et al, 2019[62]	16	XDP	bilateral GPi	VBM	BFMDRS, UPDRS III	6 months	caudate grey matter volume reductions with poorer outcomes
Gonzalez-Escamilla et al, 2019[65]	- 36<br>- 15<br>(51)	- GD<br>- CD	bilateral GPi	FreeSurfer (Graph theory)	- BFMDRS<br>- TWSTRS	≥ 3 years	cortical and subcortical volume reductions and alterations to network measures with poorer outcomes
Okromelidze et al, 2020[66]	39	idiopathic GD	bilateral GPi	OCM (NC)	UDRS	≥ 6 months<br>R: 6 – 24 months	connectivity with widespread cortical regions associated with directional outcomes
Raghu et al, 2021[67]	19	isolated CD	bilateral posterior GPi	OCM (PT)	TWSTRS	M: 12.05 months<br>R: 6 – 18 months	increased pallidal connectivity to M1-putamen associated with greater outcomes
Tremor							

Klein et al, 2012[77]	- 9<br>- 3<br>(12)	- ET<br>- tPD	- bilateral VIM<br>- bilateral VIM<br>(1 unilateral)	OCM (PT)	FTM	R: 4 – 12 weeks	connectivity to cerebellum, subcortical and motor cortical regions associated with outcomes
Coenen et al, 2014[74]	- 6<br>- 2<br>- 3<br>(11)	- ET<br>- MDT/ET<br>- tPD	- bilateral VIM<br>- bilateral VIM<br>- unilateral VIM	proximity (DT)	FTM	9-months<br>R: 3 – 17 months	closer contact proximity to DRTT associated with greater tremor reduction
Groppa et al, 2014[76]	- 2<br>- 5<br>(7)	ET	- unilateral VIM<br>- bilateral VIM	OCM (PT)	FTM	M: 19.7 ± 14.4 months	increased connectivity with dentate nucleus associated with greater outcomes
Schlaier et al, 2015[81]	5	ET	bilateral VIM	proximity (DT)	unstandardised	intra-operative	no effect of contact proximity to the DRTT for tremor alleviation
Anthofer et al, 2017[73]	6	ET	bilateral VIM	proximity (DT)	unstandardised	R: 10 – 52 months	closer contact proximity to DRTT associated with greater tremor reduction
Al-Fatly et al, 2019[72]	36	ET	bilateral VIM/ZI	OCM (NC)	FTM	≥ 3 months, M: 12 ± 9.86 months	cerebellothalamocortical connectivity associated with outcomes
Dembek et al, 2020[75]	13	ET	bilateral PSA/VIM	proximity (NC)	FTM	2 months	closer contact proximity to DRTT associated with greater tremor reduction
Petry-Schmelzer et al, 2020[79]	7	ET	bilateral PSA/VIM	overlap (PT)	FTM	3 months	larger VTA overlap with DRTT associated with greater outcomes
Riskin-Jones et al, 2021[80]	14	ET	unilateral, bilateral VIM	OCM (PT)	FTM	≥ 4 months<br>M: 405.22 d	effective contacts connected to PCG and brainstem/cerebellum
Middlebrooks et al, 2021[78]	- 83<br>- 14<br>(97)	ET	unilateral VIM/VOp border	OCM (NC)	FTM	- 6.8 ± 1.5 months<br>- 7.1 ± 1.9 months	increased connectivity with sensorimotor cortices, via DRTT, associated with greater outcomes
Tsuboi et al, 2021[82]	- 20<br>- 20<br>(40)	- ET<br>- DyT	unilateral VIM/VOp border	overlap and OCM (NC)	FTM	- 6.6 ± 1.8 months<br>- 6.8 ± 2.2 months	DRTT and pallidothalamic tracts importance in DyT and ET

[a]Numbers in brackets reflect the total patient sample in the study. [b]Where stated, follow-up periods are reported as mean (M) ± standard deviation and range (R). Abbreviations: akinetic-rigid Parkinson's disease, AR-PD; caudal zona incerta, cZi; cervical dystonia, CD; dentate nucleus, DN; dentatorubrothalamic tract, DRTT; deterministic tractography, DT; diagnosis, DX; diffusion weighting imaging, DWI; dystonic tremor, DyT;

outcome connectivity mapping, OCM; essential tremor, ET; fractional anisotropy, FA; generalised dystonia, GD; globus pallidus internus, GPi; myoclonic dystonic tremor, MDT; nigrostriatal pathway, NSP; normative connectome, NC; nucelus basalis of Myenert, nbM; Parkinson's disease, PD; pedunculopontine nucleus, PPN; posterior subthalamic area, PSA; postural instability and gait disorder, PIGD; precentral gyrus, PCG; primary motor cortex, M1; primary somatosensory cortex, S1; probabilistic tractography, PT; quantitative susceptibility mapping, QSM; subthalamic nucleus, STN; superior cerebellar peduncle, SCP; superior frontal gyrus, SFG; supplementary motor area, SMA; tremor dominant Parkinson's disease, tPD; ventral intermediate thalamus, VIM; ventralis oralis posterior, VOp; volume of tissue activated, VTA; voxel-based morphometry, VBM; X-Linked Dystonia Parkinsonism, XDP; zona incerta; ZI

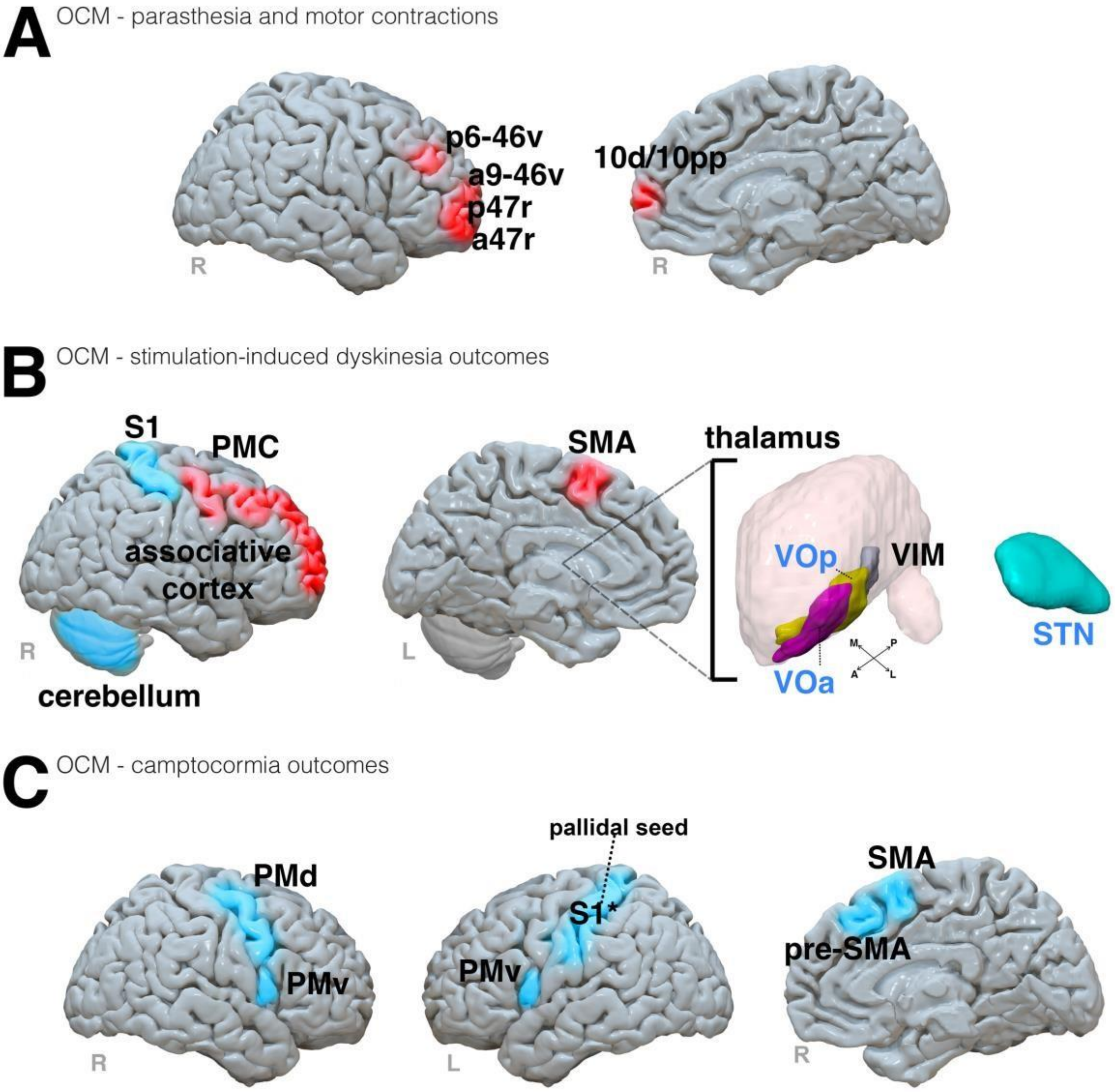

Supplementary Figure 1 Outcome connectivity mapping for non-cardinal symptoms in Parkinson's disease (A) brain regions associated with paraesthesia and motor contractions side effects in patients with Parkinson's disease. **(B)** brain regions associated with stimulation induced dyskinesia. **(C)** brain regions associated with camptocormia symptom improvement in patients with Parkinson's disease. *Association with S1 is resultant from pallidal DBS implantation and tractography seeding. All else is from STN implantation and seeding. Brain regions involved in symptom improvement and worsening are highlighted in blue and red, respectively.
